# Aconitate decarboxylase 1 mediates the acute airway inflammatory response to environmental exposures

**DOI:** 10.3389/fimmu.2024.1432334

**Published:** 2024-09-16

**Authors:** Aaron D. Schwab, Amy J. Nelson, Angela M. Gleason, Oliver W. Schanze, Todd A. Wyatt, Dhananjay D. Shinde, Peng Xiao, Vinai C. Thomas, Chittibabu Guda, Kristina L. Bailey, Tammy Kielian, Geoffrey M. Thiele, Jill A. Poole

**Affiliations:** ^1^ Division of Allergy & Immunology, Department of Internal Medicine, College of Medicine, University of Nebraska Medical Center, Omaha, NE, United States; ^2^ Division of Pulmonary, Critical Care and Sleep Medicine, Department of Internal Medicine, College of Medicine, University of Nebraska Medical Center, Omaha, NE, United States; ^3^ Veterans Affairs Nebraska-Western Iowa Health Care System, Research Service, Omaha, NE, United States; ^4^ Department of Environmental, Agricultural and Occupational Health, College of Public Health, University of Nebraska Medical Center, Omaha, NE, United States; ^5^ Department of Pathology, Microbiology, and Immunology, University of Nebraska Medical Center, Omaha, NE, United States; ^6^ Department of Genetics, Cell Biology and Anatomy, University of Nebraska Medical Center, Omaha, NE, United States; ^7^ Division of Rheumatology & Immunology, Department of Internal Medicine, College of Medicine, University of Nebraska Medical Center, Omaha, NE, United States

**Keywords:** ACOD1, organic dust, endotoxin, environmental health, immunometabolism, macrophages, inhalation

## Abstract

**Background:**

Environmental lipopolysaccharide (LPS) and microbial component-enriched organic dusts cause significant lung disease. These environmental exposures induce the recruitment and activation of distinct lung monocyte/macrophage subpopulations involved in disease pathogenesis. Aconitate decarboxylase 1 (*Acod1*) was one of the most upregulated genes following LPS (vs. saline) exposure of murine whole lungs with transcriptomic profiling of sorted lung monocyte/macrophage subpopulations also highlighting its significance. Given monocyte/macrophage activation can be tightly linked to metabolism, the objective of these studies was to determine the role of the immunometabolic regulator ACOD1 in environmental exposure-induced lung inflammation.

**Methods:**

Wild-type (WT) mice were intratracheally (i.t.) instilled with 10 μg of LPS or saline. Whole lungs were profiled using bulk RNA sequencing or sorted to isolate monocyte/macrophage subpopulations. Sorted subpopulations were then characterized transcriptomically using a NanoString innate immunity multiplex array 48 h post-exposure. Next, WT and *Acod1^−/−^* mice were instilled with LPS, 25% organic dust extract (ODE), or saline, whereupon serum, bronchoalveolar lavage fluid (BALF), and lung tissues were collected. BALF metabolites of the tricarboxylic acid (TCA) cycle were quantified by mass spectrometry. Cytokines/chemokines and tissue remodeling mediators were quantitated by ELISA. Lung immune cells were characterized by flow cytometry. Invasive lung function testing was performed 3 h post-LPS with WT and *Acod1^−/−^* mice.

**Results:**

*Acod1^−/−^* mice treated with LPS demonstrated decreased BALF levels of itaconate, TCA cycle reprogramming, decreased BALF neutrophils, increased lung CD4^+^ T cells, decreased BALF and lung levels of TNF-α, and decreased BALF CXCL1 compared to WT animals. In comparison, *Acod1^−/−^* mice treated with ODE demonstrated decreased serum pentraxin-2, BALF levels of itaconate, lung total cell, neutrophil, monocyte, and B-cell infiltrates with decreased BALF levels of TNF-α and IL-6 and decreased lung CXCL1 vs. WT animals. Mediators of tissue remodeling (TIMP1, MMP-8, MMP-9) were also decreased in the LPS-exposed *Acod1^−/−^* mice, with MMP-9 also reduced in ODE-exposed *Acod1^−/−^* mice. Lung function assessments demonstrated a blunted response to LPS-induced airway hyperresponsiveness in *Acod1^−/−^* animals.

**Conclusion:**

*Acod1* is robustly upregulated in the lungs following LPS exposure and encodes a key immunometabolic regulator. ACOD1 mediates the proinflammatory response to acute inhaled environmental LPS and organic dust exposure-induced lung inflammation.

## Introduction

Of the 12.6 million deaths that result from unhealthy environments every year, 8.2 million are caused by non-communicable diseases mostly originating from inhaled exposure (1). Industrial and agricultural intensification synergistically elevates worker and non-worker risk for adverse respiratory health outcomes (2–5). Biologic material use in agricultural, waste treatment, recycling, and food production work settings elevates the risk of inhaling harmful bioaerosols, particularly organic dust (6, 7). Organic dusts are complex, heterogeneous collections of particle-associated bacterial and fungal components that provoke lung inflammatory responses (8). An immunogenic component of many disease-causing environmental dusts is lipopolysaccharide (LPS) or endotoxin, a membrane component of gram-negative bacteria (9, 10). Despite advances in identifying respirable hazards and understanding the key signaling pathways involved in mediating the lung inflammatory response, there remains a paucity of knowledge and efficacious therapeutic options to accelerate recovery and prevent disease progression.

Recruited and activated lung monocyte/macrophage subpopulations induced by environmentally sourced organic dust extract (ODE) and LPS have been identified as central mediators of tissue damage, inflammation, and fibrosis as well as resolution and recovery processes (11–19). Specifically, lung monocyte/macrophage subpopulations have been implicated in driving the transition from acute lung inflammation to tissue recovery, with the initiation of prefibrotic processes having potential long-term, adverse health outcomes (11, 20, 21). Whereas the interplay between lung monocyte/macrophage function and metabolic plasticity is appreciated in pulmonary infection, sepsis, and chronic lung disease, its role in inhaled environmental exposure-induced lung injury is unknown (22–26). As monocyte/macrophage recruitment and activation is tightly linked to cellular metabolism, studying the immunomodulatory role of metabolic enzymes and their bioactive metabolites represents a new avenue for mechanistic characterization and potential therapy development (27).

An emerging immunometabolic regulator of activated monocytes/macrophages is aconitate decarboxylase 1 (ACOD1). ACOD1 is a multifunctional regulator of infection and inflammation responsible for itaconate metabolism and is found predominately in the mitochondria (28, 29). The biological implications of ACOD1 are becoming increasingly nuanced as it plays context-dependent roles in the regulation of both pro- and anti-inflammatory responses after induction by inflammatory stimuli (30). In various inflammatory and infectious contexts, *Acod1*-deficient mice have exhibited more robust proinflammatory cytokine burst, aberrant neutrophil infiltration, and worsened survival (29). Conversely, ACOD1 induction can promote virus replication, exacerbate ROS-mediated tissue damage, induce immune paralysis, and cause ferroptosis-mediated cell death (31). In the context of lung disease, itaconate has been shown to mitigate pulmonary fibrosis severity, interferon responses in influenza A infection, and pulmonary *Brucella* proliferation and infection (32–34). ACOD1/itaconate deficiency has also been shown to exacerbate endotoxemia-induced acute lung injury via the inhibition of autophagy (35). Additionally, *Acod1* deletion augmented urban dust particulate matter (PM)-induced macrophage production of IL-6 and IL-1β although mouse lung inflammation following an *in-vivo* PM exposure was not affected by ACOD1 (36).

We sought to characterize transcriptomic changes at the whole lung and monocyte/macrophage subpopulation levels to identify critical mediators of exposure-induced lung inflammation. These transcriptomic investigations demonstrated robust *Acod1* upregulation, prompting us to determine the role of ACOD1 in mediating the lung inflammatory response and resolution processes following inhaled, environmentally relevant inflammatory exposures. We used *Acod1*-deficient (knockout) mice to characterize the relative changes in inflammatory responses, carbohydrate metabolism, pathology, tissue repair, and airway hyperresponsiveness (AHR) that occur in the absence of ACOD1. Elucidation of the role of ACOD1 in this model may inform novel therapeutic strategies for environmental exposure-induced lung disease.

## Materials and methods

### Environmental exposure agents: LPS and ODE

LPS from gram-negative *Escherichia coli* (O55:B5; Sigma, St. Louis, MO) and an aqueous ODE prepared from swine confinement feeding facilities (17) served as the two inhalant exposure agents. Briefly, settled surface dust (1 g) was incubated in sterile Hank’s Balanced Salt Solution (10 mL; Mediatech, Manassas, VA) for 1 h and centrifuged for 30 min at 2,850×*g* twice, with the final supernatant filter-sterilized (0.22 um) to remove microorganisms and coarse particles. Stock ODE was batch prepared and stored at −20°C; aliquots were diluted for each experiment to a final concentration (vol/vol) of 25% in sterile phosphate-buffered saline (PBS, pH 7.4; diluent). The rationale for the use of LPS is that it is commercially available and elicits dose-dependent, reproducible proinflammatory lung responses in humans and rodents that could be translated to various environmental exposures. Agricultural ODE represents a “real-life” complex organic dust exposure. Endotoxin concentrations were determined using the limulus amebocyte lysate assay (Lonza, Walkersville, MD). Endotoxin levels averaged 1.308–2.616 μg (~10–50 EU) for 25% ODE. Prior mass spectrometry studies of ODE have revealed significant amounts of muramic acid (peptidoglycan marker) and 3-hydroxy fatty acids (endotoxin marker), but not ergosterol (fungi marker) as compared to house dust (17).

Mean or median concentrations of endotoxin in ambient air generally fall in the range of 0.006–5.7 EU/m^3^ (2). Polluted urban environments, however, can achieve endotoxin concentrations as high as 75 EU/m^3^ (37). Agricultural practices elevate endotoxin concentrations where concentrated animal feeding operations (CAFOs) can have endotoxin concentrations ranging from <1 to 4,153 EU/m^3^ with upwind ambient concentrations averaging 22.8 EU/m^3^ and downwind ambient concentrations averaging 65.3 EU/m^3^ (38). Our 10-μg LPS dose is used to model an acute, high-concentration exposure to a bacterial component-enriched environment, whereas our ODE exposure is intended to model a complex exposure to organic dust comprised of an endotoxin concentration comparable to real-life exposure.

### Animal exposure model

For transcriptomic investigations, C57BL/6 mice (between 6 and 8 weeks of age) were purchased from The Jackson Laboratory (Bar Harbor, ME), randomized upon arrival, and allowed to acclimate for 1 week prior to initiation of experiments (note: the authors A.J.N. and A.G. and facility staff were aware of the randomization, whereas all the other authors were blinded). Male mice were utilized for all transcriptomic studies because we have previously demonstrated that female mice were less susceptible to inflammatory agent inhalation-induced airway and systemic inflammatory effects (39). Airway inflammation was induced using a singular intratracheal instillation whereby mice were lightly sedated under the continual flow of 1.5% isoflurane (VetOne, Boise, ID) and received treatment with either 50 μL of sterile saline or 10 μg of LPS.

For studies exploring the role of ACOD1 in environmental exposure-induced lung inflammation, C57BL/NJ6 (WT) and C57BL/6NJ-*Acod1^em1(IMPC)J^*/J (*Acod1^−/−^)* mice between 6 and 8 weeks of age were purchased from The Jackson Laboratory (Bar Harbor, ME). In the latter strain (#029340; RRID: IMSR_JAX:029340), the aconitate decarboxylase 1 (*Acod1*) encoding gene was knocked out by means of CRISPR technology [Knockout Mouse Phenotyping Program (KOMP^2^) at The Jackson Laboratory]. The *Acod1^−/−^* mice have normal development without adverse phenotypic differences relative to WT mice. Both male and female mice were utilized for these studies comparing WT and *Acod1^−/−^* animal responses to environmental exposures to capture potential sex-mediated differences. Mice were lightly sedated under isoflurane (VetOne, Boise, ID) and received one treatment with 50 μL of LPS (10 μg) or ODE (25%) (8). Control (CXN) mice represent WT (C57BL/NJ6) mice intratracheally (i.t.) instilled with sterile saline. An intubation laryngoscope (Harvard Apparatus, Holliston, MA) enabled tracheal visualization and access to the intratracheal instillation technique. Weights were recorded daily, and all animals were euthanized 48 h after the acute environmental agent exposure by isoflurane followed by exsanguination (right axillary blood collection). Our previous work identified peak monocyte/macrophage lung recruitment and activation at 48 h (2 days) post-LPS exposure with corresponding elevations in inflammatory markers and resolution of most inflammatory indices by 1 week (15). Similar findings were also reported with organic dust extract exposure (18). Thus, to adequately capture differences in cellular recruitment and inflammatory mediator prevalence, we utilized this 2-day timepoint. No respiratory distress, signs of stress, or significant weight loss (defined as >20%) was observed throughout this period.

### Fluorescence-activated cell sorting of monocyte/macrophage subpopulations

In three independent studies with two to four mice pooled per treatment group (LPS or saline, respectively) and following removal of blood from pulmonary vasculature, lungs were inflated with 1 mL of digestion solution/mouse containing 0.5 mg/mL of Liberase™ (Millipore Sigma, St. Louis, MO) and 235.5 U/mL of DNAse I in Hank’s Balanced Salt Solution (pH = 7.2). Lung cells were dissociated with a gentleMACS dissociator (Miltenyi Biotech, Auburn, CA) and incubated for 15 min at 37°C in a shaking incubator. Digestion solution activity was neutralized with FA3 buffer (10 mM of HEPES, 2 mM of EDTA, 1% FBS in PBS). The single-cell lung suspensions were incubated with CD16/32 (Fc Block, Biolegend, San Diego, CA) to minimize non-specific antibody staining. Next, cells were stained with monoclonal antibodies (mAbs) directed against rat anti-mouse CD45 (clone 30-F11; BD Biosciences, Franklin Lakes, NJ), Ly6C (clone AL-21, BD Biosciences), Ly6G (clone 1A8, BD Biosciences), CD11b (clone M1/70, BD Biosciences), and hamster anti-mouse CD11c (clone N418, Invitrogen, Eugene, OR) and with LIVE/DEAD Fixable Blue Dead Cell Stain kit (Invitrogen, Eugene, OR). Flow sorting was performed by FACSAria II (BD Biosciences). To remove lymphocytes and neutrophils, live CD45^+^ singlets were reverse gated on lymphocytes [characteristic forward scatter-area (FSC-A) × side scatter-area (SSC-A)] and neutrophils (Ly6C^+^Ly6G^+^ cells) to select for the following monocyte/macrophage populations: CD11c^+^CD11b^lo^ alveolar (Alv) macrophages (Mɸ), CD11c^+^CD11b^+^ activated (Act) Mɸ, CD11c^int^CD11b^+^ recruited/transitioning monocyte (Mono)-Mɸ, and CD11c^−^CD11b^+^ Mono. Sal Alv Mɸ and Sal Mono were sorted from the saline treatment group, whereas LPS Act Mɸ, LPS Mono-Mɸ, and LPS Mono were sorted from the LPS-treated group. This gating strategy for these five monocyte/macrophage populations is consistent with previous reports by us and others (40–43).

### RNA extraction

Total RNA was extracted from homogenized mouse whole lung cells or from each sorted lung monocyte/macrophage subpopulation single-cell suspension using RNeasy Mini Kit according to the manufacturer’s instructions (Qiagen, Germantown, MD). RNA samples were analyzed with respect to purity and potential degradation in the UNMC Genomics Core Facility using a NanoDrop (Thermo Scientific, Nanodrop Products, Wilmington, DE) instrument to measure absorbance. Potential degradation of the sample was assessed by analysis of the RNA using an Advanced Analytical Technical Instrument Fragment Analyzer (AATI, Ames, IA). All samples had A260/280 of 1.8 or above and RQN scores >8.0.

### Whole lung RNA-sequencing and analysis

Libraries were generated using 1 μg of total RNA from each sample and the NuGEN Universal Plus mRNA-Seq library kit from TECAN (Redwood City, CA). Libraries were multiplexed and sequenced on the NextSeq550 Sequencer (Illumina) to generate a total of approximately 20 to 25 million 75 bp paired reads for each sample. The original fastq format reads were trimmed by the fqtirm tool (https://ccb.jhu.edu/software/fqtrim) to remove adaptors, terminal unknown bases (Ns), and low-quality 3′ region (Phred score < 30). The trimmed fastq files were processed by FastQC (44) for quality control. The trimmed fastq files were then processed by a standard pipeline utilizing STAR (45) as the aligner and RSEM (46) as the tool for annotation and quantification at the gene level. The raw counts were used for differential expressed gene (DEG) analysis by the R/Bioconductor package DESeq2 (47). The reads were mapped to the mm10 (GRCm38) mouse reference genome. The resulting *p*-values from each comparison were adjusted for false discovery rate (FDR) using the Benjamini–Hochberg (B-H) method (48). The threshold for significant DEGs was B-H-adjusted *p*-value (padj) <0.05. The heatmap was plotted by pheatmap 1.0.12 package in R 4.0.3 based on the value of log_2_(TMP_value+0.0001) for all significant genes (adj *p* < 0.05) in all samples to avoid any nonsense values of log_2_(0). The heatmap then underwent symmetric normalization to improve comparative visualization. The volcano plots were created using the GraphPad Prism software, version 10.2.2 (GraphPad, San Diego, CA), with statistical significance accepted at *p <*0.05. Gene enrichment analyses were performed using Ingenuity Pathway Analysis (IPA; Qiagen Inc., https://www.qiagenbioinformatics.com/products/ingenuity-pathway-analysis). The R package *GOPlot* was utilized to visualize the relationship between genes and enriched pathways. The datasets have been deposited to the Gene Expression Omnibus (GEO) database with access number GSE267022.

### NanoString^®^ nCounter expression analysis of monocyte/macrophage subpopulations

For transcriptomic analysis of the three independent experiments of the fluorescence-activated cell sorting (FACS)-isolated five monocyte/macrophage subpopulations from two to four pooled animals per treatment group (saline and LPS) per experiment, the NanoString nCounter system was utilized. The Mouse Myeloid Innate Immunity profiling panel containing 770 genes (NanoString, Seattle, WA) was utilized and run according to the manufacturer’s instructions. Sequencing libraries were generated by the UNMC NGS Core beginning with 500 ng of total RNA from each sample using the NuGEN Universal Plus mRNA-Seq library kit from TECAN (Redwood City, CA) following the manufacturer’s recommended procedure. Resultant libraries were assessed for size of the insert by analysis of an aliquot of each library on a Bioanalyzer instrument (Agilent Technologies, Santa Clara, CA). Each library had a unique indexing identifier barcode allowing the individual libraries to be multiplexed together for efficient sequencing. Multiplexed libraries were sequenced on a single 150-cycle mid-output flow cell of the NextSeq550 Sequencer (Illumina) using a 2 × 75-bp paired-end protocol to generate a total of approximately 50 million pairs of reads for each sample. The nSolver Analysis Software (NanoString, Seattle, WA) was utilized to compute differential gene expression between two subpopulations. Expression data were normalized to 20 housekeeping genes.

### Blood collection and serum

Whole blood was collected from the axillary artery at euthanasia, and serum was harvested as previously described (49). Serum pentraxin-2 (murine acute-phase reactant protein) levels were assessed using a Quantikine ELISA kit (R&D, Minneapolis, MN), according to the manufacturer’s instructions [minimal detection difference (MDD) of 0.159 ng/mL].

### Lavage fluid cells and lung homogenates

Bronchoalveolar lavage fluid (BALF) was collected from each animal with three 1 mL aliquots of sterile phosphate-buffered saline (PBS, pH 7.4). Total BALF cell counts from pooled lavages were enumerated using a BioRad TC 20 cell counter. Differential cell counts were determined from cytospin-prepared slides (Cytopro Cytocentrifuge, ELITech Group, Logan, UT) with Diff-Quick (Siemens, Newark, DE). Cell-free BALF from the first lavage fraction was evaluated for cytokines and chemokines by murine-specific enzyme-linked immunosorbent assays (ELISA). After BALF isolation and removal of blood from pulmonary vasculature, lung tissue homogenates were prepared by homogenizing lung samples (one-half of each right lung) in 500 μL of sterile PBS. The levels of tumor necrosis factor (TNF)-α, transforming growth factor (TGF)-β, IL-6, IL-10, and the murine neutrophil chemoattractant CXCL1 were quantitated by ELISA (R&D Systems) following the manufacturer’s instructions. The kits had MDD of 1.88, 31.3, 1.6, 31.3, and 2.0 pg/mL for TNF-α, TGF-β, IL-6, IL-10, and CXCL1, respectively. Interferon (IFN)-γ was quantitated by ELISA (Invitrogen) with an MDD of 0.7 pg/mL following the manufacturer’s instructions. Additionally, lung tissue homogenates were assessed for regulators of extracellular matrix deposition including matrix metalloproteinase (MMP)-3 and tissue inhibitor of metalloproteinase (TIMP)-1 (ELISA; R&D Systems; MDD of 0.125 and 0.031 ng/ml, respectively) as well as MMP-8 and MMP-9 (ELISA; Abcam, Boston, MA; MDD of 0.053 and 0.078 ng/ml, respectively).

### Metabolomics: LC-MS/MS analysis of TCA cycle metabolites

To prepare samples, 200 μL of cell-free BALF was added to 1 mL of chilled 80% methanol (LC-MS grade, Thermo Fisher) (13). C_3_-pyruvate and ^13^C_4_-succinate were used as the internal standards and spiked in samples before metabolite extraction. Samples were subsequently centrifuged at 13,000 rpm for 10 min at 4°C. The resulting supernatant was then transferred to a new tube, dried in a SpeedVac (6.5 h, 30°C), and then held at −80°C until metabolomics analysis. Dried samples were reconstituted in 100 µL of 50% methanol. An ultra-performance liquid chromatography *I*-class system (Waters, USA) connected to a triple-quadrupole-ion trap hybrid mass spectrometer (QTRAP6500+, Sciex, USA) was used for the separation and subsequent detection of metabolites of interest. Separation of these metabolites was performed by liquid chromatography using an Acquity UPLC CSH Phenyl-Hexyl column (100 × 2.1 mm ID; 1.7 µm particle size) analytical column procured from Waters, USA, and a binary solvent system with a flow rate of 0.4 mL/min. A CSH Phenyl-Hexyl guard column (20 × 2.1 mm ID; 1.7 µm particle size, Waters) was connected before the analytical column. Mobile phase A was composed of 0.1% formic acid in LC-MS-grade water, whereas mobile phase B was 0.1% formic acid in 100% LC-MS-grade acetonitrile. The column was maintained at 50°C, and the autosampler temperature was maintained at 5°C. The injection volume of each sample was 5 µL, and a total of 500 µL of weak wash solvent comprising 10% aqueous methanol and 500 µL of strong wash solvent comprising 100% acetonitrile were used after each injection. The QTRAP6500 + instrument was operated in polarity switching mode for targeted quantitation of tricarboxylic acid (TCA) cycle metabolites through a multiple reaction monitoring (MRM) process. Electrospray ionization parameters were optimized as follows: electrospray ion voltages of −4,500 and 5,500 V in negative and positive modes, respectively; source temperature of 400°C; curtain gas of 35; and gases 1 and 2 of 40 and 40 psi, respectively. Compound-specific parameters were optimized for each compound using manual tuning. These parameters include the MRMs (Q1/Q3), declustering potentials (DPs), and collision energies (CEs) for each metabolite. These details are listed as follows: lactate (Q1/Q3, DP, CE: 89.0/43.0, −53, −16), citric acid (191.0/111.0, −40, −17.6), pyruvate (87.0/32.0, −46, −14), succinate (117/73, −32, −14), malate (133.0/115.0, −55, −15.6), fumarate (115.0/71.0, −80, −13), α-ketoglutaric acid (145.0/101.0, −40, −13.8), aconitate (173.0/85.0, −40, −17.3), and itaconate (131.0/85.1, 40, 16.8). For each metabolomics run, missing values were estimated by one-third of the minimum positive value of each metabolite. All metabolite values were standardized to CXN mice (*n* = 5) metabolite average values and therefore represented as fold change relative to CXN.

### Lung and lung draining lymph node cell staining and flow cytometry

Lung cell infiltrates were determined following lung cell dissociation from the remaining one-half of each right lung lobe as previously described (49). Pulmonary lymph nodes were harvested from each mouse following published laboratory precedent (50), and a single-cell suspension was achieved by using our previously described protocol for lung dissociation and single-cell suspension (49). For immune cell characterization, cells were stained with fluorophore-conjugated monoclonal antibody against rat anti-mouse CD45 (clone 30-F11, BD Biosciences), CD11b (clone M1/70, BD Biosciences), Ly6G (clone 1A8, BD Biosciences), CD11c (clone N418, Invitrogen), CD4 (clone RM4-5, BD Biosciences), CD8 (clone 53-6.7, BD Biosciences), CD19 (clone 1D3, Invitrogen), CD24 (clone M1/69, BioLegend), hamster anti-mouse CD3e (clone 145-2C11, BD Biosciences), CD103 (clone 2E7, Thermo Fisher), and mouse anti-mouse NK1.1 (clone PK136, BD Biosciences). Cells were then evaluated on a BD LSRII YG (Green Profile) cytometer. In each case, a minimum of 50,000 events were acquired and analyzed for each sample. Post-acquisition, all flow cytometry data were exported and stored using the flow cytometry standard (FCS) 3.1 format and subsequently analyzed using FlowJo software version 10.10.0 (FlowJo, Ashland, OR). The gating strategies for lung Ly6G^+^ neutrophils, CD11c^+^CD11b^lo^ Alv Mɸ, CD11c^+^CD11b^+^ Act Mɸ, CD11c^int^CD11b^+^ Mono-Mɸ, and CD11c^−^CD11b^+^ Mono, CD3^+^CD4^+^ T cells, CD3^+^CD8^+^ T cells, CD19^+^ B cells, and NK cells were performed as previously reported (**Supplementary Figure 1**) (15, 39, 51–54). In a separate experiment, whole lungs of LPS-exposed WT (*n* = 5) and *Acod1^−/−^* (*n* = 5) mice were harvested 48 h post-LPS exposure, homogenized, and stained to characterize dendritic cells via flow cytometry. Briefly, after the removal of debris, doublets, dead cells, CD45^−^ cells, lymphocytes, neutrophils, and CD11b^+^ cells, CD11c^+^ cells were quadrant gated by CD24 and CD103 expression to delineate CD103^+^ and CD103^−^CD24^+^ dendritic cells (55). The gating strategies for lymph node Ly6G^+^ neutrophils, Ly6C^hi^CD11b^hi^ monocytes, CD11c^hi^CD11b^variable^ macrophages, CD3^+^CD4^+^ T cells, CD3^+^CD8^+^ T cells, CD19^+^ B cells, NK cells, and dendritic cells were informed by previously reported work (**Supplementary Figure 2**) (55–57). The percentage of all respective lung cell populations was determined from live CD45^+^ lung leukocytes after excluding debris and doublets. This percentage was multiplied by the respective total lung cell numbers to determine specific cell population numbers for each animal. Lymph node-specific cell populations are represented by the percent of CD45^+^ cells where immune cell numbers are standardized to the CD45^+^ cell number per biologic sample and multiplied by 100.

### Lung myeloid cell functional assays

In separate studies, phagocytic ability and reactive oxygen species (ROS) production were determined for lung monocytes/macrophages and neutrophils from whole lung cells of the *in-vivo* LPS-exposed WT and *Acod1^−/−^* mice. At 48 h post-exposure, single lung cell suspensions were incubated for 30 min at 37°C with 25 μM of CellROX Deep Red (Invitrogen, Carlsbad, CA) or with opsonized, fluorescein-conjugated *E. coli* BioParticles (Invitrogen, Carlsbad, CA) to quantify ROS and phagocytic activity according to the manufacturers’ instructions, respectively. Cells were then placed on ice and incubated as described above for markers indicative of monocytes, macrophages, and neutrophils (i.e., live/dead, CD45, CD11b, CD11c, Ly6G). Cells were subsequently washed with cold PBS, fixed with 4% paraformaldehyde, and analyzed on a BD LSRII YG (Green Profile). The gating strategy for the neutrophils and monocyte/macrophage subpopulations is consistent with that shown in **Supplementary Figure 1**. Lung Ly6G^+^ neutrophils, CD11c^+^CD11b^lo^ alveolar macrophages, CD11c^+^CD11b^+^ activated macrophages, CD11c^int^CD11b^+^ monocytes–macrophages, and CD11c^−^CD11b^+^ monocytes were analyzed for CellROX- and BioParticles-associated fluorescence as previously described (18). Data are represented by the percent of the cell population that exhibited BioParticles- (%BioParticles+) or CellROX-specific (%CellROX+) probe fluorescence. Additionally, mean fluorescence intensity (MFI) was quantified and compared per cell population between WT and *Acod1^−/−^* mice.

### Lung histopathology and Masson’s modified trichrome staining

Left lungs were excised and inflated to 15 cm H_2_O pressure with 10% formalin (Fisher Scientific, Fair Lawn, NJ) for 24 h to preserve pulmonary architecture as previously described (49). The fixed lobes were then placed into cassettes, embedded in paraffin, cut (to 4–5 μm) at midpoint sections to include regions of both large and small airways as well as blood vessels, and stained with hematoxylin and eosin (H&E). Slides were then reviewed at all scanning magnifications by an experimental pathologist blinded to the treatment conditions and semiquantitatively assessed for the degree and distribution of lung inflammation. Using a previously published scoring system, each lung was given an inflammatory score value from 1 to 4 (a higher score indicating greater inflammatory changes in the lung) (58). Lung sections were also stained with modified Masson’s trichrome and scanned with an Aperio scanner (Leica Biosystems, Deer Park, IL) by the institution’s Tissue Sciences Core Facility. Aperio ImageScope Software (Leica Biosystems, Deer Park, IL) was utilized to export lung sections at full resolution in TIFF format. Collagen staining of the whole lung images was isolated with ImageJ FIJI software (version: 2.9.0/1.53t U.S. National Institutes of Health, Bethesda, MD) and the Colour Deconvolution plugin (59) using methods to create a user-defined color matrix as described by the plugin authors. The integrated density of the isolated collagen staining was then measured using a static thresholding scheme as previously described (15).

### Invasive pulmonary function measurement

Altered lung function is a hallmark characteristic of lung inflammation (60). Although AHR is a canonical feature of allergic asthma, AHR can also be induced by ozone exposure, viral infection, lipopolysaccharide, and agricultural exposure (61–64). Baseline airway resistance and compliance as well as AHR was invasively assessed by direct airway resistance, 3 h post-i.t.-instilled LPS or saline, using a computerized small-animal ventilator (FinePointe, Buxco Electronics, Wilmington, NC), as previously described (65). Dose responsiveness to aerosolized methacholine (0–48 mg/mL) was obtained and is reported as total lung resistance (R_L_). Our previous work and others have demonstrated peak AHR at 3–5 h post-LPS enriched exposures with loss of AHR 24 h post-exposure (63, 65, 66), informing the 3-h timepoint for AHR testing.

### Statistical analysis

Sample-size requirements were extrapolated from a previous work assessing post-LPS lung recovery treatments in C57BL/6 (15). The mean (± SD) of CD11c^int^CD11b^hi^ transitioning/recruited Mono-MФ was 0.26 × 10^5^ (0.09 × 10^5^) with saline and 6.5 × 10^5^ (2.2 × 10^5^) with LPS treatment 48 h post-exposure; thus, a sample size of *n* = 2 in each group would achieve 80% power at the 0.05 level of significance to determine an influx of these cells following inflammatory agent exposure as compared to saline control. Littermates and purchased mice were used to achieve the following sample size per treatment group: *n* = 5 (5 male WT, Saline; referred to as CXN), *n* = 19 (9 male and 10 female WT, LPS), *n* = 18 (8 male and 10 female *Acod1^−/−^*, LPS), *n* = 17 (7 male and 10 female WT, ODE), and *n* = 19 (9 male and 10 female *Acod1^−/−^*, ODE). Numbers less than the maximum number reflect limitations in the available sample quantity or quality. Data are presented as the mean ± standard error of the mean (± SEM) with scatter plots depicted for each data point. The Shapiro–Wilk test was utilized to test for normality among treatment groups. If the normality condition was satisfied, a parametric statistical test (one-way ANOVA with subsequent Tukey’s multiple comparison test) was used, and if not satisfied, a non-parametric statistical test (Kruskal–Wallis with subsequent Dunn’s multiple comparison test) was used to assess differences between any two groups. All statistical analyses were performed using GraphPad Prism (version: 10.2.2) software, and statistical significance was accepted at a *p*-value <0.05 unless otherwise specified.

### Ethics statement

Neither human participants, data, nor tissues were used in these experiments. The study was conducted and reported in accordance with ARRIVE guidelines (https://arriveguidelines.org). All animal procedures were approved by the University of Nebraska Medical Center (UNMC) Institutional Animal Care and Use Committee and were in accordance with NIH guidelines for the use of rodents.

## Results

### Bioinformatical analysis of the whole lung transcriptome following lung-delivered LPS

**Figure 1:**
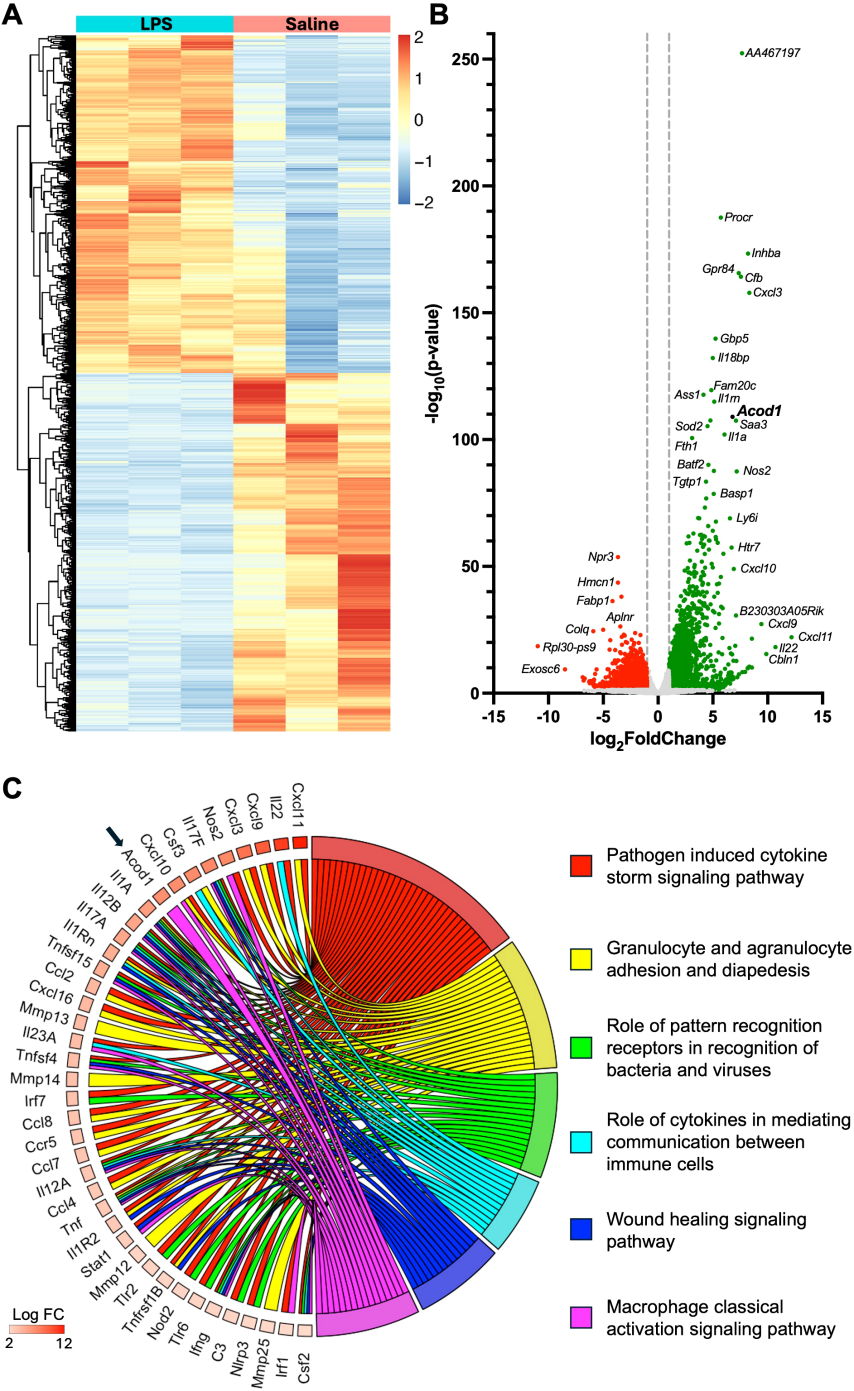
Comparative transcriptome of lung tissue from lipopolysaccharide (LPS)- and saline-exposed mice at 48 h post-exposure. **(A)** Heatmap demonstrates unsupervised hierarchical clustering of samples (*n* = 3 per treatment) and relative frequencies of genes subjected to symmetric normalization of log_2_(transcripts per million [TPM] + 0.0001) for all significant genes (adj *p* < 0.05) to avoid any nonsense values. The color scheme represents symmetric normalization of relative frequencies from 2 (red, high expression) to −2 (blue, low expression). **(B)** Volcano plot demonstrates statistical significance (−log_10_(*p*-value)) vs. magnitude of change (log_2_FoldChange) in the expression of specified gene transcripts (−log_10_(*p*-value) > 1.3) with green reflecting upregulated and red reflecting downregulated genes. **(C)** Chord plot demonstrates the top canonical pathways of the whole lung transcriptome based on the Ingenuity Pathway Analysis (IPA) output and corresponding adjusted *p*-value (adj *p* < 0.05) and the top upregulated genes (*p* < 0.05) associated with each modulated pathway.

To identify unique regulatory transcripts and potential therapeutic targets following inhalant LPS exposure, bulk RNA sequencing (RNA-seq) of whole lung tissue processed from saline-treated (*n* = 3) and 10 µg of LPS-treated (*n* = 3) animals was examined 48 h after challenge. Of the 20,749 genes identified, 6,515 demonstrated significant changes (*p* < 0.05) in expression between treatment groups. There were 3,163 genes that were significantly upregulated, whereas 3,352 genes were significantly downregulated. Differential expression analysis (DEA) was performed by comparing the whole lung transcriptome between LPS- and saline-exposed mice with unsupervised hierarchical clustering delineating these distinct transcriptomes (**Figure 1A**). A volcano plot shows differential gene expression achieving statistical significance (*p* < 0.05) with its magnitude of fold change (log_2_FoldChange > 1) in LPS-exposed relative to saline-exposed mice. The top canonical pathways (padj < 0.05) and their top enriched genes (*p* < 0.05) in mice exposed to LPS are demonstrated in the chord plot (**Figure 1C**). *Acod1* was significantly upregulated (28th most upregulated transcript of 3,163 significantly upregulated genes) in these comparative transcriptomic investigations (**Figure 1B**) and defined as a driver of the macrophage classical activation signaling pathway (**Figure 1C**). IPA of the whole lung transcriptome implicated pathways canonically associated with inflammatory cell recruitment, immune cell signaling, wound healing, and classical macrophage activation following lung exposure to LPS (**Figure 1C**).

### LPS exposure induces transcriptional reprogramming in the lung monocyte/macrophage compartment

**Figure 2:**
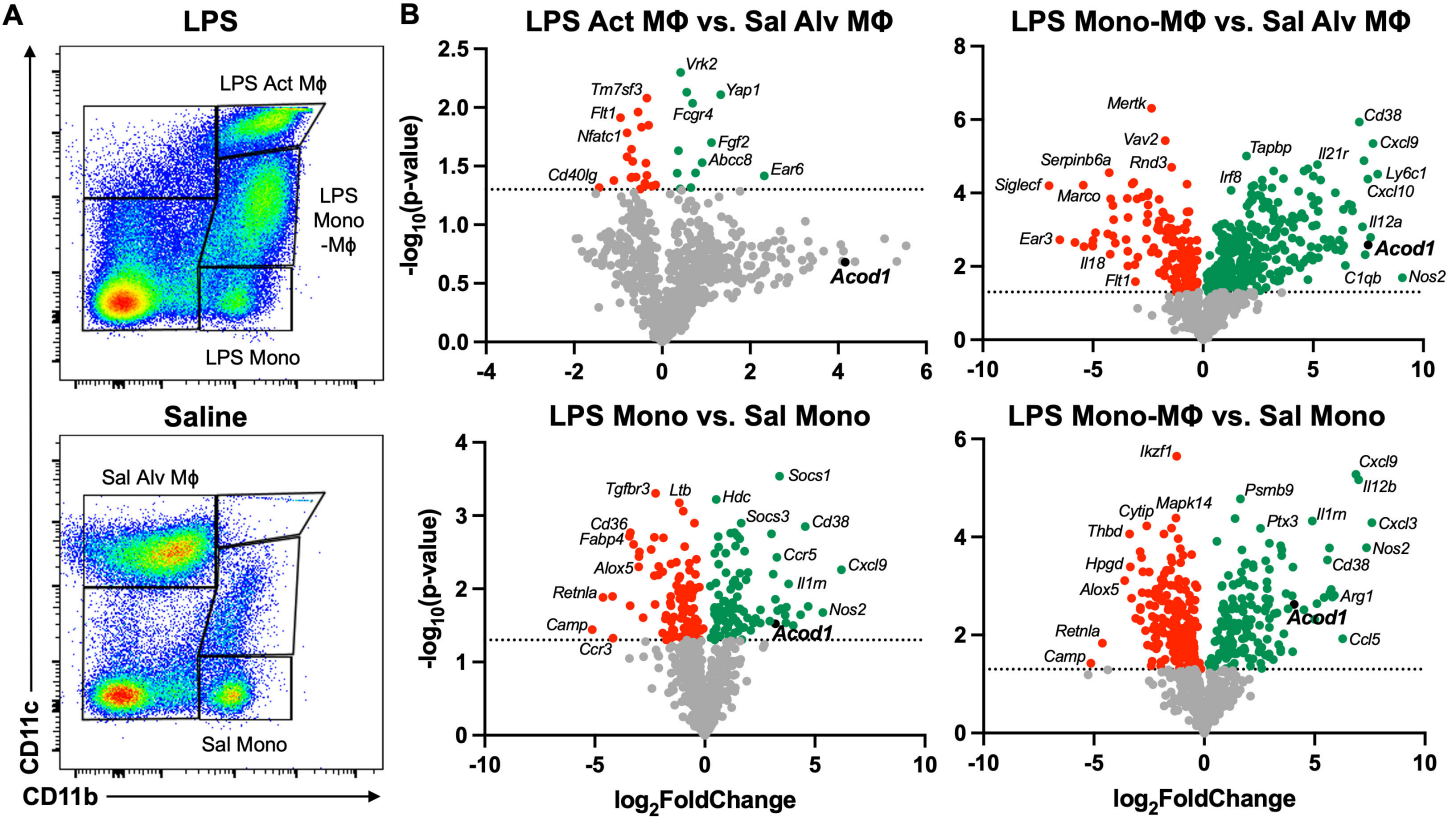
Acute exposure to LPS differentially modulates myeloid cell gene transcription with notable *Acod1* upregulation. **(A)** Representative image of gates for the five lung monocyte (Mono)/macrophage (MΦ) subpopulations: Saline (Sal) Alveolar (Alv) MΦ: CD11c^+^CD11b^lo^, LPS Activated (Act) MΦ: CD11c^+^CD11b^hi^, Transitioning LPS Mono-MΦ: CD11c^int^CD11b^hi^, and Sal and LPS Mono: CD11b^hi^CD11c^−^ after exclusion of debris, doublets, dead cells, CD45^−^ cells, lymphocytes, and neutrophils. **(B)** Volcano plots demonstrate statistical significance (−log_10_(*p*-value) vs. magnitude of change (log_2_FoldChange) in the expression of specified gene transcripts. Statistical significance is denoted by the dotted line (−log_10_(*p*-value) > 1.3). *n* = 3 samples per lung monocyte/macrophage subpopulation for differential gene expression analysis.

To characterize transcriptomic changes specific to lung monocyte/macrophage subpopulations post-LPS exposure, we defined the differential gene expression of distinct populations of infiltrating lung cells. Lung monocyte/macrophage subpopulations were FACS-isolated based on relative CD11b and CD11c surface expression of live, singlet, CD45^+^ cells that were neither neutrophils nor lymphocytes (**Figure 2A**). Consistent with previous studies (15), at 48 h post-LPS exposure, “resting” alveolar macrophages (referred to as Sal Alv MФ) are “lost” as they upregulate CD11b and now exhibit an activated phenotype (referred to as LPS Act MФ). The LPS-activated monocyte/macrophage subpopulations (LPS Act MФ, LPS Mono-MФ, and LPS Mono) demonstrated distinctive transcriptomic profiles relative to saline-exposed subpopulations (Sal Alv MФ and Sal Mono) with universal upregulation of regulatory transcripts across LPS-exposed subpopulations (**Figure 2B**). There were inadequate numbers of Sal Mono-MФ for experimental studies. To further elucidate the characteristics of the LPS-induced Mono-MФ subpopulation, its transcriptomic profile was compared to both Sal Alv MФ and Sal Mono (**Figure 2B**). *Acod1* was universally upregulated (log_2_FoldChange) in the LPS Act MФ vs. Sal Alv MФ (3.2), LPS-Mono-MФ vs. Sal Alv MФ (7.49), and LPS Mono vs. Sal Mono (4.07) subpopulations but did not reach statistical significance (*p* > 0.05) with the LPS Act MФ vs. Sal Alv MФ comparison. The LPS Act MФ vs. Sal Alv MФ comparison yielded the most distinctive upregulated gene profile, given there was no overlap between these top 10 most upregulated genes and the top 10 most upregulated genes across the remaining three comparisons.

The top 5 most significantly (*p* < 0.05) upregulated genes for the LPS Act MФ vs. Sal Alv MФ comparison included *Ear6* (2.32), *Yap1* (1.33), *Fgf2* (1.12), *Abcc8* (0.91), and *Cd70* (0.76). There were three gene transcripts that were among the top 10 most upregulated across all remaining comparisons (LPS Mono vs. Sal Mono, LPS Mono-MФ vs. Sal Alv MФ, and LPS Mono-MФ vs. Sal Mono): *Nos2* (5.36, 9.05, and 7.35, respectively), *Il12a* (4.35, 7.24, and 5.8), and *Ccl5* (3.68, 7.36, and 6.27). In comparison to LPS Mono vs. Sal Mono and LPS Mono-MФ vs. Sal Alv MФ, both *CD38* (log_2_FoldChange: 4.56 and 7.09, respectively) and *Ccl12* (3.71 and 7.6) were among the top 10 most significantly upregulated transcripts. In the LPS Mono vs. Sal Mono and LPS Mono-MФ vs. Sal Mono comparisons, *Cxcl9* (6.21 and 6.87), *Il12b* (4.02 and 6.99), and *Cxcl3* (3.65 and 7.59) were among the top 10 most significantly upregulated gene transcripts, respectively. Gene transcripts that were unique to a comparison’s top 10 most significantly upregulated genes are as follows: LPS Mono vs. Sal Mono: *Cxcl11* (4.7) and *Il1rn* (3.81); LPS Mono-MФ vs. Sal Alv MФ: *Ly6c1* (7.93), *Cxcl9* (7.72), *Acod1* (7.49), *Cxcl10* (7.48), and *Aoah* (7.31); LPS Mono-MФ vs. Sal Mono: *Arg1* (5.89), *Il1a* (5.75), *Cxcl16* (5.7), and *Ccl22* (5.67). The distinctive transcriptomes among the monocyte/macrophage subpopulations emphasize the heterogeneity of the lung monocyte/macrophage compartment and identify potential regulatory targets that merit further investigation. *Acod1* is of particular interest given that it is highly upregulated at both the whole lung and monocyte/macrophage resolution. Given the cellular specificity of ACOD1 to monocytes/macrophages, modulation of this regulatory molecule will mitigate off-target effects and alter the functionality of these cellular populations, which are integral to disease pathogenesis (67). As such, the following studies focused on characterizing the role of ACOD1 in environmental exposure-induced lung inflammation.

### *Acod1* depletion reduces LPS- and ODE-induced serum pentraxin-2 levels but not LPS- or ODE-induced weight loss

**Figure 3:**
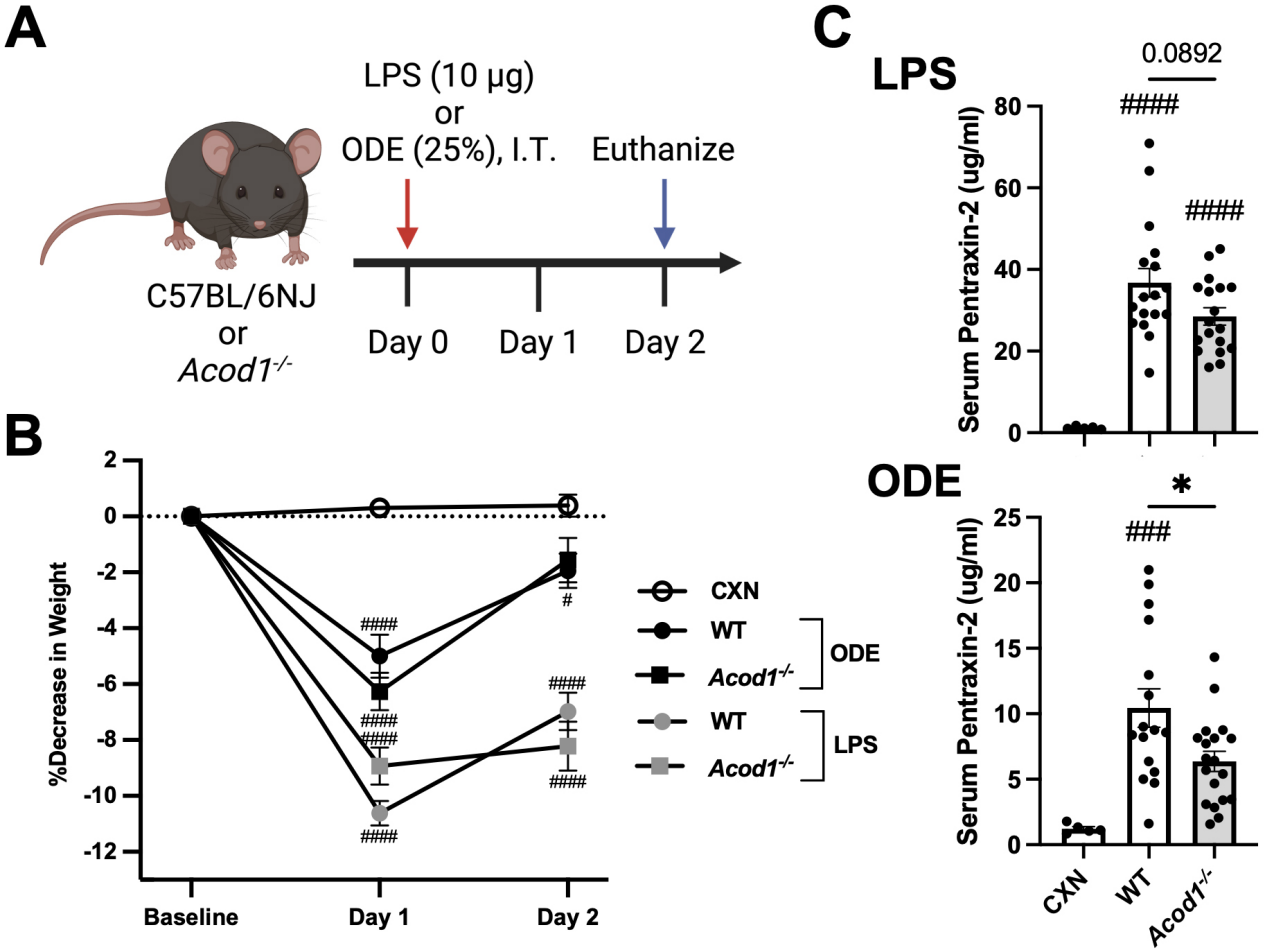
*Acod1* depletion decreases ODE-induced serum pentraxin-2 levels but not LPS- or ODE-induced weight loss. **(A)** Schematic of the experimental design (created with BioRender.com). **(B)** Line graph depicts the mean with SEM bars of percent changes in weight over time. **(C)** Scatter plot graphs depict the mean with SEM bars of serum pentraxin-2 levels among treatment groups. *n* = 5 (CXN), *n* = 17–19 (8–9 male and 9–10 female WT mice, LPS), *n* = 18 (8 male and 10 female *Acod1^−/−^* mice, LPS), *n* = 16–17 (7 male and 9–10 female WT mice, ODE), and *n* = 19 (9 male and 10 female *Acod1^−/−^* mice, ODE). Statistical significance vs. CXN (#*p* < 0.05, ###*p* < 0.001, ####*p* < 0.0001); between groups (*p < 0.05).

To characterize the functional role of ACOD1 in mediating the inflammatory response to a one-time, lung-targeted inflammatory agent (LPS and ODE), C57BL/6NJ (WT) and C57BL/6NJ-*Acod1^em1(IMPC)J^*/J (*Acod1^−/−^)* mice were i.t. instilled with either LPS (10 μg), 25% ODE, or saline (Control; CXN) with endpoints collected 48 h post-exposure (schematic, **Figure 3A**). Weights were collected to assess the systemic response to inhaled environmental exposures, and serum pentraxin-2, a murine acute-phase reactant protein, was quantified to assess systemic responsiveness to inflammatory stimuli. Mouse serum pentraxin-2 is produced by hepatocytes and induced by IL-6 (similar to human C-reactive protein), making it an appropriate biomarker representative of non-specific, systemic inflammation (68). LPS- and ODE-induced weight loss was not dependent on *Acod1* (**Figure 3B**). However, LPS- and ODE-induced serum pentraxin-2 was decreased in the *Acod1^−/−^* mice, reaching significance (*p* < 0.05) for ODE-WT vs. *Acod1^−/−^* mice but not LPS-WT vs. *Acod1^−/−^* animals (**Figure 3C**).

### ACOD1 alters carbohydrate metabolism in the airway through TCA cycle modulation

**Figure 4:**
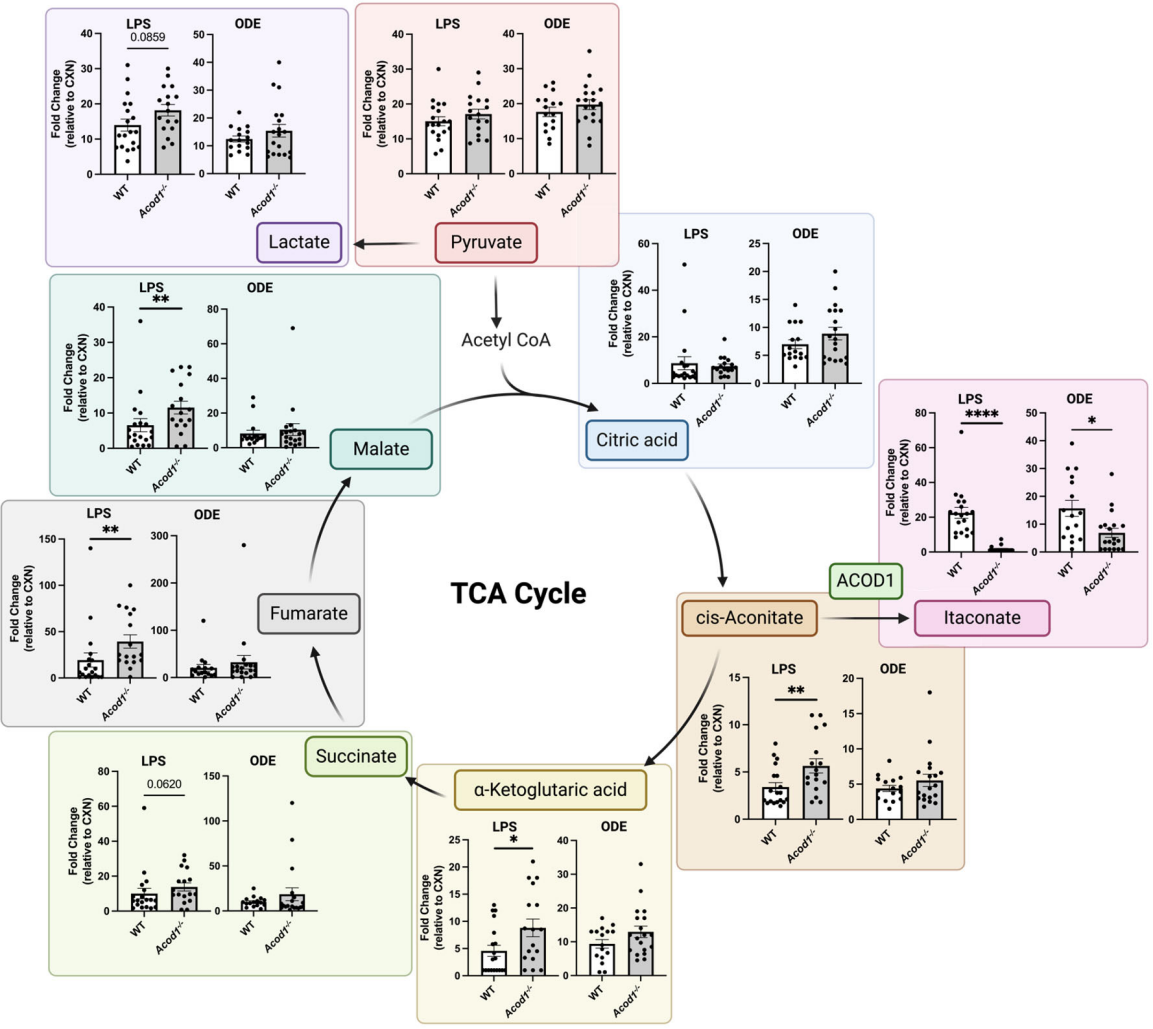
*Acod1^−/−^* mice demonstrate modulation of tricarboxylic acid cycle (TCA) intermediates following environmental exposure. A simplified schematic of the TCA cycle with scatter plot graphs depicting the mean with SEM bars between treatment groups. Graphs show the relative abundance of indicated metabolites, represented by fold change relative to CXN (saline-treated WT mice). *n* = 5 (CXN), *n* = 19 (9 male and 10 female WT mice, LPS), *n* = 18 (8 male and 10 female *Acod1^−/−^* mice, LPS), *n* = 17 (7 male and 10 female WT mice, ODE), and *n* = 19 (9 male and 10 female *Acod1^−/−^* mice, ODE). Statistical significance between groups (*p < 0.05, **p < 0.01, ****p < 0.0001).

The purpose of this targeted metabolomic investigation by mass spectrometry was two-fold: 1) validate effective *Acod1* deletion in the *Acod1^−/−^* mice by itaconate quantification and 2) characterize the downstream metabolic effects of ACOD1 removal on the TCA cycle in our model system. Relative BALF metabolite quantities are presented as fold change of LPS or ODE exposure relative to CXN (saline-treated). Itaconate levels were significantly decreased in *Acod1^−/−^* as compared to WT or *Acod1*-sufficient mice (**Figure 4**). Thus, *Acod1^−/−^* mice have sufficient ACOD1 deletion to yield observable differences in BALF itaconate concentrations following environmental inflammatory agent exposure. Metabolic disturbances downstream of ACOD1 were only identified in the LPS-exposed mice where *cis*-aconitate, α-ketoglutaric acid, fumarate, and malate were significantly increased in *Acod1^−/−^* mice as compared to WT (**Figure 4**).

### Cellular infiltrates and inflammatory mediators are dependent on ACOD1 following LPS exposure in the lung

**Figure 5:**
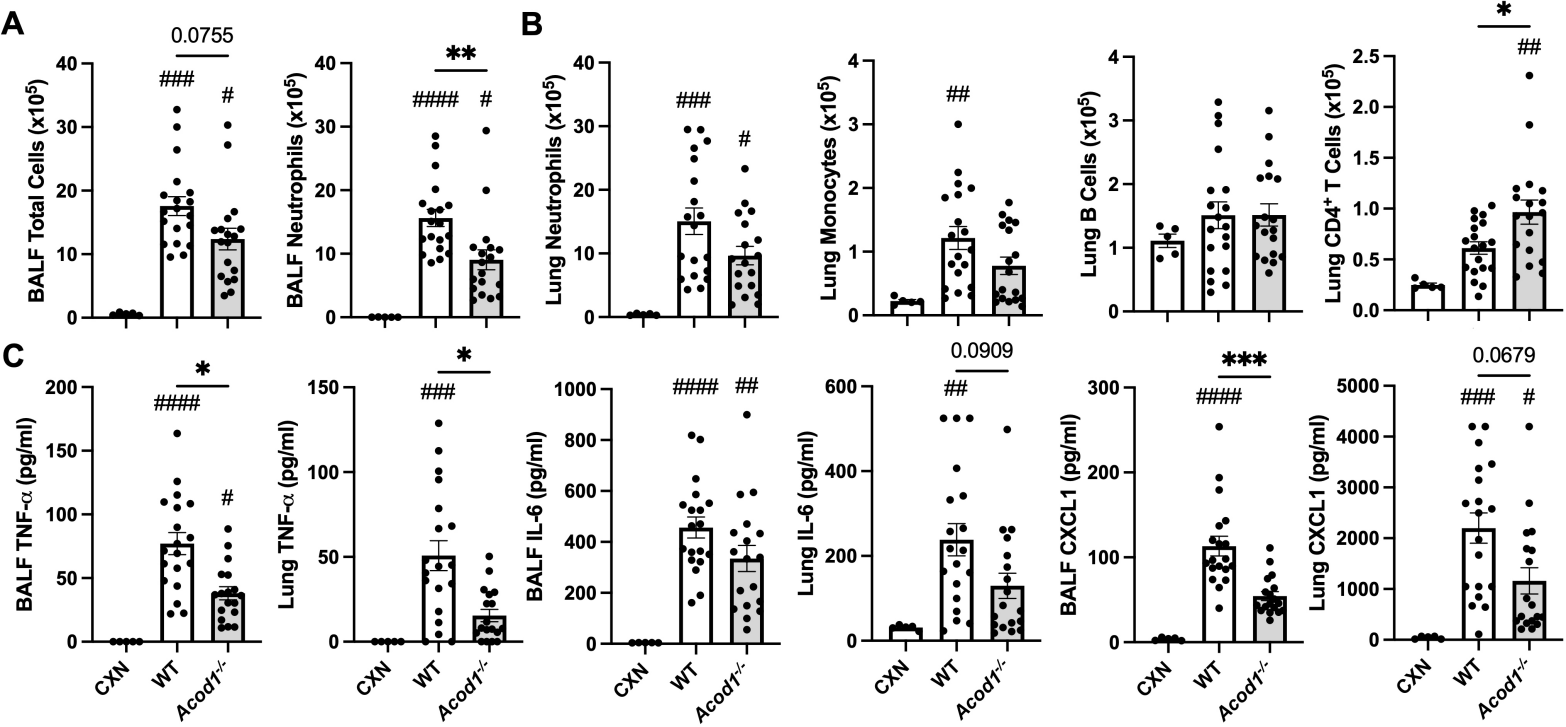
LPS-induced cellular infiltrates and inflammatory mediators are reduced in *Acod1^−/−^* mice. Scatter plot graphs depict the mean with SEM bars among treatment groups. **(A)** Total cellular influx and neutrophils in BALF. **(B)** Neutrophils, monocytes, B cells, and CD4^+^ T cells in lung tissue quantified by flow cytometry. **(C)** Levels of airway inflammatory markers determined by ELISA from BALF and lung homogenate. *n* = 5 (CXN), *n* = 19 (9 male and 10 female WT mice), and *n* = 18 (8 male and 10 female *Acod1^−/−^* mice). Statistical significance vs. CXN (#*p* < 0.05, ##*p* < 0.01, ###*p* < 0.001, ####*p* < 0.0001); between groups (*p < 0.05, **p < 0.01, ***p < 0.001).

To characterize the role of ACOD1 in mediating local, inflammatory processes, lung tissue and BALF from WT and *Acod1^−/−^* animals were harvested 48 h post-i.t. LPS and assessed for cellular composition and inflammatory indicators. In the LPS-exposed mice, BALF neutrophil influx was decreased in *Acod1^−/−^* mice relative to WT, despite no significant differences in total cells (**Figure 5A**). LPS-induced lung CD4^+^ T-cell infiltrates were decreased in *Acod1^−/−^* vs. WT animals, and there were no differences between WT and *Acod1^−/−^* mice regarding LPS-induced neutrophils, monocytes, and B-cell infiltrates (**Figure 5B**). LPS-induced BALF levels of TNF-α and CXCL1, but not IL-6, were decreased in *Acod1^−/−^* mice vs. WT mice (**Figure 5C**). Lung levels of TNF-α (but not IL-6 and CXCL1) induced by LPS were also reduced in *Acod1^−/−^* vs. WT mice (**Figure 5C**). Additional efforts to characterize the difference in LPS-induced airway inflammatory responses between WT and *Acod1^−/−^* mice that did not achieve statistical significance are included in **Supplementary Table 1**. There were no significant differences between LPS-treated WT (*n* = 5) and *Acod1^−/−^* (*n* = 5) mice regarding lung dendritic cell populations. Neither the number of CD103^+^CD24^+^ dendritic cells (WT mean ± SEM vs. *Acod1^−/−^* mean ± SEM: 0.609 ± 0.294 × 10^5^ vs. 0.344 ± 0.035 × 10^5^; *p* = 0.397) nor the number of CD103^−^CD24^+^ dendritic cells (2.98 ± 1.01 × 10^5^ vs. 1.37 ± 0.097 × 10^5^; *p* = 0.152) in whole lung samples was altered between WT and *Acod1^−/−^* mice. No significant differences in cellular composition were observed between WT and *Acod1^−/−^* pulmonary draining lymph nodes following a one-time LPS exposure (**Supplementary Table 3**). ACOD1 appears to participate in the lung proinflammatory response to LPS given the elevated inflammatory markers and cellular infiltrates in *Acod1^−/−^* vs. WT mice.

### ODE exposure-induced cellular influx and proinflammatory mediators are reduced in *Acod1^−/−^* mice

**Figure 6:**
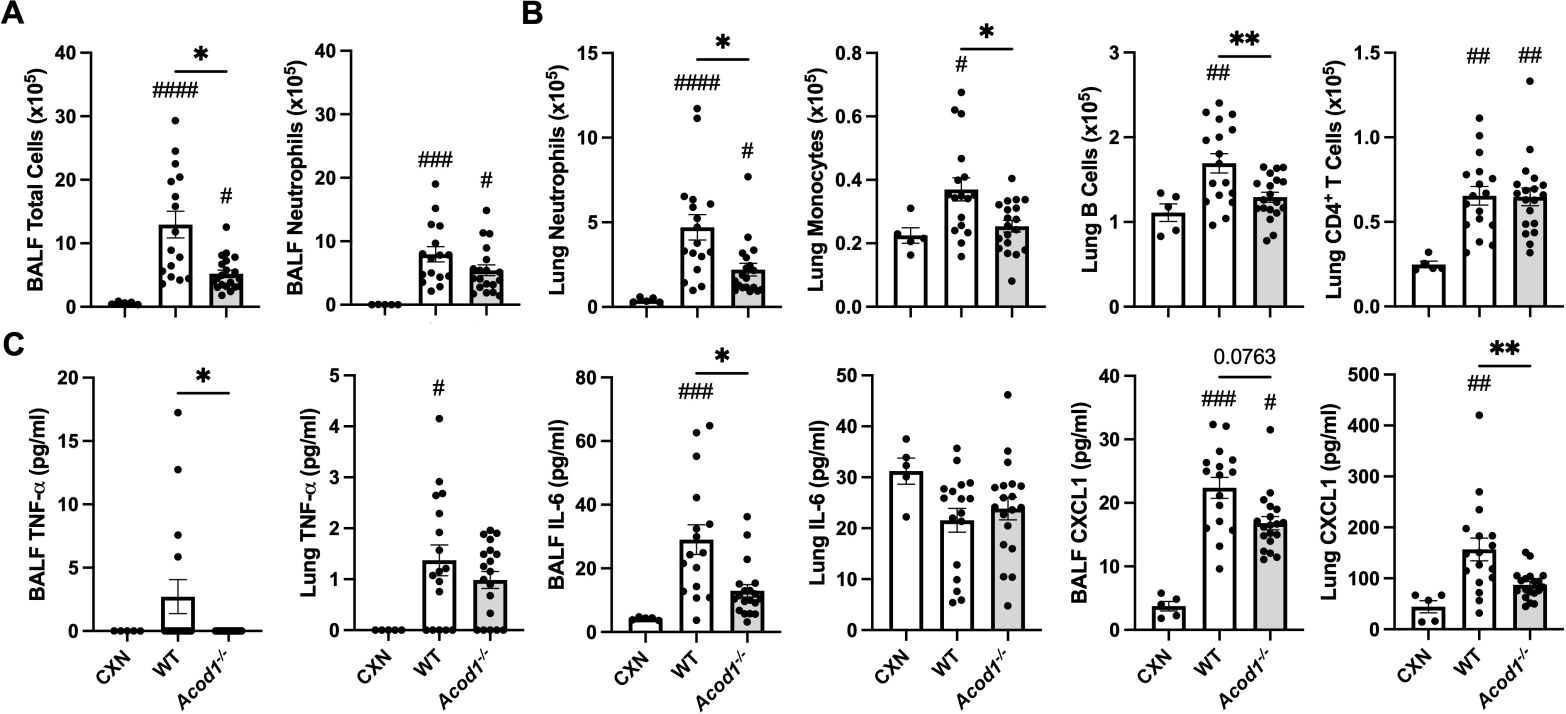
*Acod1^−/−^* mice exhibit decreased mediators of lung and airway inflammation following a one-time ODE exposure. Scatter plot graphs depict the mean with SEM bars among treatment groups. **(A)** Total cellular influx and neutrophils in BALF. **(B)** Neutrophils, monocytes, B cells, and CD4^+^ T cells in lung tissue quantified by flow cytometry. **(C)** Levels of airway inflammatory markers determined by ELISA from BALF and lung homogenate. *n* = 5 (CXN), *n* = 16–17 (7 male and 9–10 female WT mice exposed to ODE), and *n* = 19 (9 male and 10 female *Acod1^−/−^* mice exposed to ODE). Statistical significance vs. CXN (#*p* < 0.05, ##*p* < 0.01, ###*p* < 0.001, ####*p* < 0.0001); between groups (*p < 0.05, **p < 0.01).

In separate studies, the effects of ODE exposure in WT and *Acod1^−/−^* mice were also evaluated to characterize the role of ACOD1 in coordinating inflammation in response to a more complex, environmentally relevant exposure. ODE-induced total cellular influx in the BALF compartment was decreased in *Acod1^−/−^* mice relative to WT without a significant change in neutrophils (**Figure 6A**). ODE-induced lung neutrophil, monocyte, and B-cell infiltrates, but not CD4^+^ T cells, were decreased in *Acod1^−/−^* vs. WT mice (**Figure 6B**). BALF levels of ODE-induced TNF-α and IL-6 and lung levels of CXCL1 were also decreased in *Acod1^−/−^* mice relative to WT (**Figure 6C**). **Supplementary Table 2** includes additional data characterizing ODE-induced airway inflammatory consequences between *Acod1^−/−^* and WT mice that did not statistically differ. ACOD1 therefore seems necessary to coordinate the characteristic, proinflammatory lung response to ODE exposure given the elevated proinflammatory phenotype observed in *Acod1^−/−^* vs. WT mice.

### ACOD1 does not significantly alter functional characteristics of myeloid-derived subpopulations post-LPS exposure

To determine whether ACOD1 deficiency impacted the functional consequences of lung myeloid cells in the setting of *in-vivo* LPS exposure, assays capturing phagocytic activity and intracellular ROS were undertaken. The only significant difference was between WT and *Acod1^−/−^*-activated macrophages (Act Mϕ; CD11c^+^CD11b^+^) whereby *Acod1^−/−^* Act Mϕ exhibited elevated intracellular ROS as quantified by MFI of the CellROX+ population (**Table 1**). Otherwise, there were no differences in ROS production or phagocytic ability as measured by the ability to uptake bioparticles between LPS-exposed WT and *Acod1^−/−^* monocyte/macrophage subpopulations or neutrophils. Thus, despite decreased indicators of inflammation/injury in *Acod1^−/−^* mice, indices of lung myeloid cell function appear preserved across cellular populations, indicating that phagocytic ability and ROS production induced by LPS exposure are not attributable to ACOD1.

**Table 1:** Phagocytic activity and reactive oxygen species (ROS) production of lung myeloid cell subpopulations at 48 h between WT and *Acod1^−/−^* mice following a one-time inhalant exposure to LPS.

	WT	*Acod1^−/−^*
Neutrophils		
Phagocytic activity		
%BioParticle+	77.8 ± 0.886	74.6 ± 1.33
MFI	7,918 ± 259	7,783 ± 191
ROS		
%CellROX+	99.0 ± 0.0245	99.0 ± 0.0245
MFI	3,579 ± 429	3,403 ± 433
Activated macrophages		
Phagocytic activity		
%BioParticles+	81.7 ± 2.03	75.9 ± 2.58
MFI	11,521 ± 497	10,355 ± 830
ROS		
%CellROX+	99.0 ± 0.00	99.0 ± 0.00
MFI	6,193 ± 472	8,035 ± 519*
Alveolar macrophages		
Phagocytic activity		
%BioParticles+	47.9 ± 4.07	43.6 ± 1.86
MFI	6,814 ± 409	7,501 ± 489
ROS		
%CellROX+	99.1 ± 0.0200	99.1 ± 0.0200
MFI	3,018 ± 932	3,607 ± 620
Monocytes–macrophages		
Phagocytic activity		
%BioParticles+	79.9 ± 1.56	75.7 ± 1.91
MFI	10,990 ± 453	9,943 ± 422
ROS		
%CellROX+	98.8 ± 0.132	99.0 ± 0.0200
MFI	3,466 ± 446	3,478 ± 577
Monocytes		
Phagocytic activity		
%BioParticles+	65.7 ± 1.45	59.2 ± 3.22
MFI	5,786 ± 257	5,264 ± 304
ROS		
%CellROX+	98.8 ± 0.186	99.0 ± 0.0200
MFI	3,870 ± 458	4,628 ± 505

Statistical difference vs. WT (*p < 0.05) (bold). n = 5 (3 male and 2 female WT mice) and n = 5 (3 male and 2 female Acod1^−/−^ mice).

MFI, mean fluorescence intensity.

### LPS-induced mediators of tissue remodeling and AHR were dependent on ACOD1

**Figure 7:**
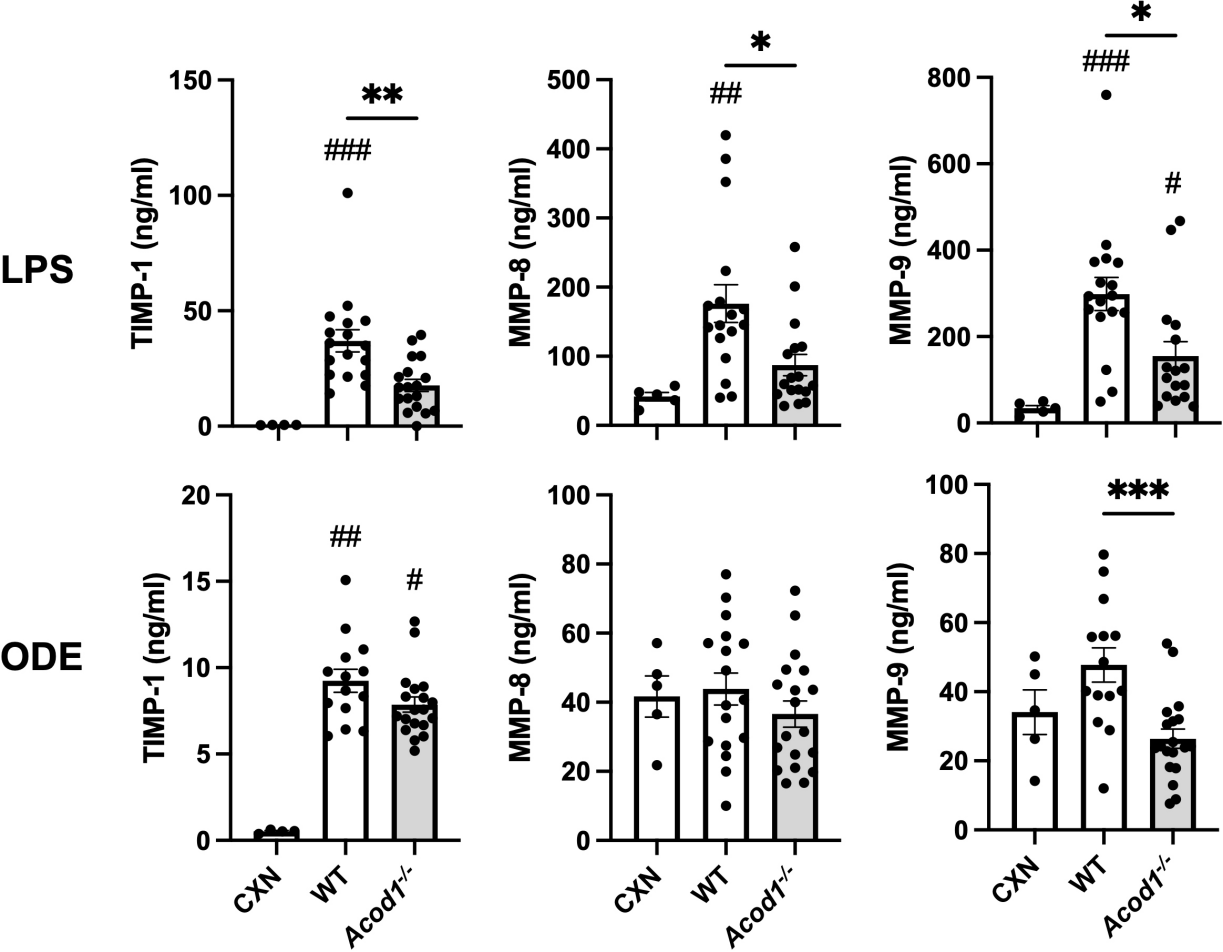
Mediators of tissue remodeling are decreased in *Acod1^−/−^* mice following environmental exposures. Scatter plot graphs depict the mean with SEM bars among treatment groups. Lung tissue levels of matrix metalloproteinases (MMPs) and tissue inhibitor of metalloproteinase (TIMP-1) are shown 48 h after a one-time LPS or ODE intratracheal instillation. *n* = 4–5 (CXN), *n* = 17 (7 male and 10 female WT mice exposed to LPS), *n* = 16–18 (6–8 male and 10 female *Acod1^−/−^* mice exposed to LPS), *n* = 17 (7 male and 10 female WT mice exposed to ODE), and *n* = 19 (9 male and 10 female *Acod1^−/−^* mice exposed to ODE). Statistical significance vs. CXN (#*p* < 0.05, ##*p* < 0.01, ###*p* < 0.001); between groups (*p < 0.05, **p < 0.01, ***p < 0.001).

**Figure 8:**
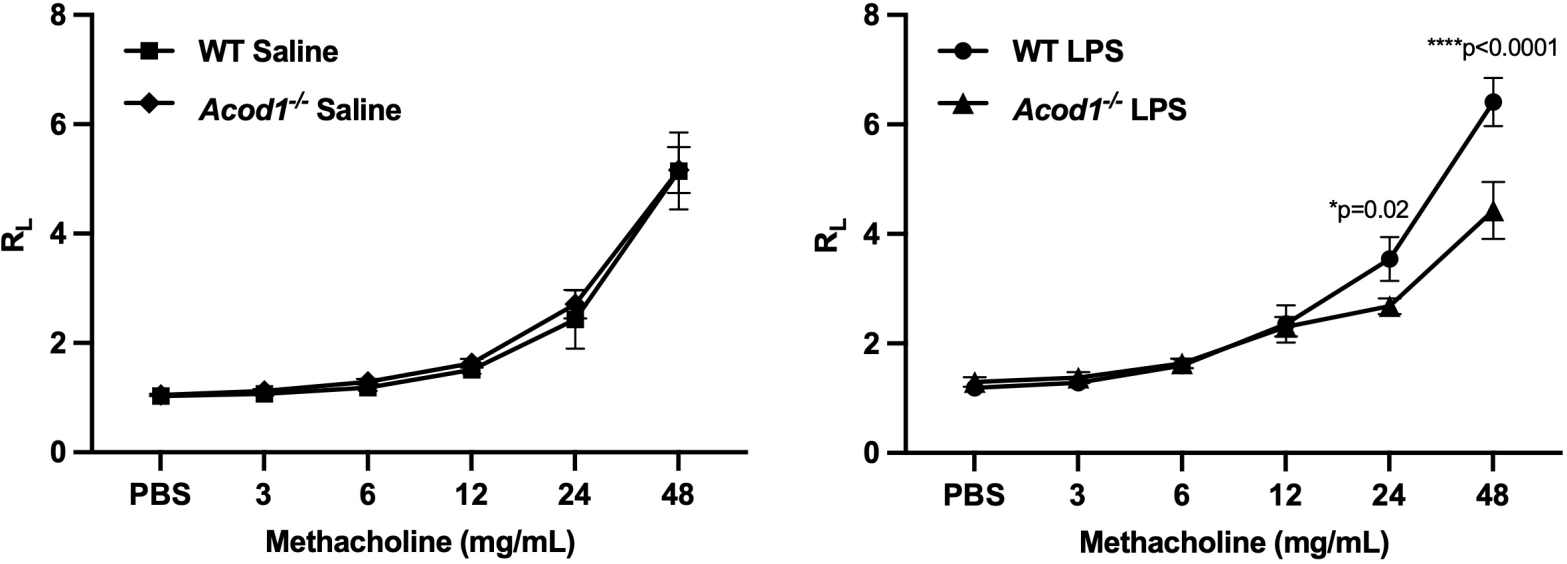
*Acod1^−/−^* mice demonstrated a blunted response to LPS-induced airway hyperresponsiveness (AHR). WT and *Acod1^−/−^* mice were initially treated with saline or LPS. Three hours following i.t. instillation, mice were tracheostomized and mechanically ventilated, and AHR to aerosolized methacholine (0 [PBS], 3, 6, 12, 24, 48 mg/mL) was measured and expressed as the mean (± SEM) total lung resistance (R_L_). Statistical difference between the LPS and saline treatment groups was determined by repeated measures of a two-way ANOVA full fit model with a two-stage linear step-up procedure of Benjamini, Krieger, and Yekutieli to control for false discovery rate. *n* = 6 male mice/group.

Other studies have demonstrated significant increases in lung MMPs during the acute phase of lung injury in animal models of lung injury resulting from LPS (by 48 h), hypoxia (peak at 72 h), hyperoxia (at 48 h), and bleomycin (by day 4) (19, 69). Given monocyte/macrophage subpopulations coordinate the transitions from acute inflammation to lung repair to aberrant fibrosis (11), we sought to determine whether ACOD1 influenced profibrotic processes and lung function at this early timepoint. LPS-induced lung levels of profibrotic mediators including TIMP-1, MMP-8, and MMP-9 were reduced in *Acod1^−/−^* vs. WT mice (**Figure 7**). Although these findings were not mirrored in ODE-exposed animals, MMP-9 was significantly reduced in *Acod1^−/−^* vs. WT mice (**Figure 7**). LPS- and ODE-induced MMP-3 did not significantly differ between *Acod1^−/−^* and WT mice (**Supplementary Tables 1**, **2**). Relative to control (CXN) treatment, LPS exposure increased the levels of TGF-β and IFN-γ, whereas ODE only increased the levels of TGF-β in WT and *ACOD1^−/−^* mice; however, there were no significant differences between LPS- or ODE-treated WT and *ACOD1^−/−^* mice (**Supplementary Tables 1**, **2**). No changes between WT and *Acod1^−/−^* mice were observed regarding histologic scoring of lung tissue sections or collagen quantification via Masson’s trichrome staining (**Supplementary Figure 3**). Acute environmental exposure-induced airway hyperresponsiveness is a phenomenon that has been observed in humans and modeled in mice (70). No differences in AHR were observed between WT and *Acod1^−/−^* mice exposed to saline (**Figure 8**). Interestingly, *Acod1^−/−^* mice displayed a significant reduction in LPS-induced AHR, compared to WT mice, at methacholine doses greater than 12 mg/mL (**Figure 8**). ACOD1 observably elevated the profibrotic and adverse functional consequences associated with an inhaled, LPS exposure.

## Discussion

LPS-enriched organic dusts are critical drivers of environmental lung inflammation and disease (71, 72). Here, our investigations first characterized the mouse lung transcriptome as well as transcriptomic changes across monocyte/macrophage subpopulations following a lung-delivered LPS exposure. Both studies demonstrated striking differences, with pathway enrichment analysis of LPS-induced whole lung transcriptomic changes implicating inflammatory cell recruitment, classical macrophage activation, cytokine-mediated signaling, pattern recognition receptor activation, and tissue repair pathways. ACOD1 expression was strikingly upregulated following LPS exposure in the whole lungs and in the following monocyte/macrophage subpopulations: LPS Act Mɸ, LPS Mono-Mɸ, and LPS Mono. Furthermore, investigations into the functional significance of ACOD1 following LPS and ODE exposures demonstrated that ACOD1 mediates proinflammatory responses and airway hyperresponsiveness to environmentally relevant exposures.

The heterogeneity of the monocyte/macrophage lung compartment is increasingly appreciated in several lung diseases, including those associated with environmental and occupational exposures. Specifically, the recruited, transitioning monocytes–macrophages have been implicated as critical cells in the immunopathogenesis of chronic lung disease with an environmental etiology (15, 73). Whereas it has also been demonstrated that depleting the recruitable reservoir of circulating monocytes results in favorable lung inflammatory consequences following LPS exposure in mice, the translational application of this approach is not infeasible. Other strategies aimed at mitigating monocyte/macrophage recruitment via targeting the CCR2 (a marker of inflammatory monocytes) failed to reduce LPS-induced airway disease in mice (19, 74). Thus, other strategies to reduce the recruitment and/or activation status of this transitioning monocyte–macrophage subpopulation are warranted.

Our monocyte/macrophage subpopulation-specific transcriptomic characterization could elucidate new targets to more specifically and robustly reduce the recruitment and/or activation of these infiltrating cells. *Ccl5* was upregulated on all LPS-activated monocyte/macrophage subpopulations and is a chemokine that primarily recruits T cells, monocytes, and dendritic cells (75). Targeted inhibition of CCR1, CCR5, or CCL5 could decrease monocyte recruitment as CCL5 mediates cellular recruitment to areas of inflammation (76, 77). Reactive nitrogen and oxygen species have been heavily implicated in environmental exposure-induced lung inflammation (78). Predictably, *Nos2* was significantly upregulated among all LPS-exposed subpopulations. NOS2 inhibitors may mitigate inflammatory consequences and associated tissue damage as a standalone or adjunctive therapy (79). *Ly6c1* was among the most upregulated transcripts in the Mono-MФ subpopulation, and Ly6C^hi^ macrophages derived from circulating Ly6C^hi^ monocytes have been well-characterized as having proinflammatory and profibrotic functions (80). Therefore, targeting this specific population of Ly6C^hi^CD11c^int^CD11b^+^ Mono-MФ may elicit therapeutic benefit by modulating the acute inflammatory response and mitigating associated fibrotic processes. Finally, *Acod1* was a distinguishing transcript of the LPS Mono-MФ, especially relative to Alv MФ, and moreover, it was the 28th most upregulated transcript in the LPS-exposed lung transcriptome. These observations informed our subsequent efforts to characterize the role of ACOD1 in mediating exposure-induced lung inflammation.

We discovered that ACOD1 is capable of mediating differential metabolic consequences depending on the complexity and composition of the environmental exposure (i.e., LPS or ODE exposure). Both the LPS and ODE exposures demonstrated decreased levels of itaconate in the absence of ACOD1; however, only the LPS-exposed mice exhibited altered carbohydrate metabolism downstream of ACOD1 where relative abundances of *cis*-aconitate, α-ketoglutaric acid, fumarate, and malate were increased in *Acod1^−/−^* mice relative to WT. Elimination of the ACOD1-mediated carbohydrate shunt in *Acod1^−/−^* mice likely resulted in corresponding increases in downstream metabolites only in LPS-exposed mice. There are potential explanations that could account for the difference in LPS- and ODE-induced metabolomic effects. First, LPS was a stronger inducer of tightly linked macrophage activity and metabolism. Next, non-LPS components of the highly complex ODE may be modulating immunometabolic perturbations. Namely, previous findings have demonstrated that the endotoxin component in swine barn dust does not completely explain the immune inflammatory response observed in ODE-exposed animals and cultured monocytes and macrophages (81). The predominance of gram-positive bacterial components, such as muramic acid, in addition to several elemental compounds, such as iron, was found to exist in the dust and likely skew the resultant inflammatory responses and phagocyte functionality (8, 81). Additionally, scant fungal and bacterial components in the dust may offset the immune paralysis caused by ACOD1; for example, β-glucan treatment has been found to inhibit LPS-induced ACOD1 expression and restore macrophage immune activity through the recovery of succinate dehydrogenase (SDH) expression (82). Alternative carbon sources capable of rescuing metabolic reprogramming or agents capable of modulating the inflammatory response are intrinsic to the complexity of the ODE exposure and likely account for the exposure-specific differences observed in our metabolomic studies.

ACOD1 was initially identified in 1995 as a bacterial LPS-inducible gene involved in innate immunity in mouse macrophages (83). Recently, cyclin-dependent kinase 2 (CDK2) was identified as a key regulator of ACOD1 expression in mouse and human monocytes and macrophages. CDK2 phosphorylation mediates the activation of mitogen-activated protein kinase 8 (MAPK8) which enables JUN-dependent transcription of *Acod1* (84). Although elucidation of the underlying mechanisms explains how inflammatory stimuli upregulate *Acod1* transcription and translation, recent advances in describing the dual role of the ACOD1–itaconate pathway in mediating inflammatory responses complicate its functional categorization. Currently, ACOD1 is considered as an immunometabolic regulator exerting a nuanced, context-dependent role where transcriptional activation of ACOD1 enables the catalysis of itaconate which can either directly or indirectly exert anti-inflammatory or proinflammatory effects (30). Preclinical studies using animal models of acute endotoxemia identified a protective effect of ACOD1 in mitigating lethal inflammation in sepsis, identifying the importance of ACOD1 expression in the antimicrobial armory of macrophages (31, 85). However, in a mouse study utilizing a cecal ligation-induced polymicrobial sepsis model, ACOD1 upregulation was responsible for the activation of immune pathways and sustained proinflammatory signaling through itaconate-dependent and independent mechanisms (84). The results of our current studies align with this latter study, where ACOD1 is important in facilitating a robust, proinflammatory response.

LPS- and ODE-induced upregulation of ACOD1 was associated with proinflammatory consequences induced with environmental exposure, and moreover, ACOD1 depletion resulted in protective benefits. Although the list of the known anti-inflammatory effects of ACOD1 is robust, knowledge related to the proinflammatory effects mediated by ACOD1 is rapidly expanding (30). For example, ACOD1 is capable of directly mediating ROS production which induces IL-1β, IL-6, IL-18, TNF, and CCL2 production (86). ACOD1 can also directly bind the GTPase, IMAP family member 7 (GIMAP7) and subsequently activate TNF pathways in isolation of itaconate production (84). Itaconate can inhibit aconitase 1 and 2 activity resulting in mitochondrial ROS (mROS) production and increased intracellular free iron, respectively (87). Both the resultant mROS and excess cellular iron-induced ROS production activate the NLRP3 inflammasome, which leads to CASP1-dependent IL-1β production (87). In contrast to our findings, others demonstrated that *Acod1^−/−^* animals had increased inflammatory/fibrotic consequences in a model of bleomycin-induced pulmonary fibrosis, suggesting that ACOD1 was important in limiting profibrotic and tissue remodeling processes (32). Intriguingly, in an LPS-induced endotoxemia model, induction of ACOD1 was found to reduce TNF production (88, 89), which also deviates from our findings in an inhaled exposure model. These diverging results support the dual nature of the ACOD1/itaconate immunometabolic axis and highlight the importance of the 1) nature of the primary niche interfacing with the exposure (systemic vs. tissue-targeted administration), 2) exposure duration (i.e., acute, repetitive, time until euthanization post-exposure), and 3) exposure character (i.e., infectious, sterile, degree of complexity) when considering the role of ACOD1 in inflammation.

We performed functional assessments of myeloid-derived lung cells to ascertain whether the clearance ability of these innate immune cells was preserved or not in the absence of ACOD1 in the setting of LPS exposure. Our results demonstrated that *Acod1^−/−^* LPS-activated macrophages exhibited increased intracellular ROS with no other significant changes across myeloid populations regarding intracellular ROS production and phagocytic activity, indicating preservation of functional indices. LPS is known to polarize macrophages toward an M1-like phenotype which have characteristically high ROS (90). Itaconate, the bioactive metabolite of ACOD1, has been identified to limit M1 polarization in macrophages (91); therefore, our observation that *Acod1^−/−^* LPS-activated macrophages exhibited increased intracellular ROS is consistent with previous studies. However, it remains unknown whether ACOD1 impacts bacterial clearance in the setting of environmental exposures, an important area for future study. These future experiments will be complicated however, given multiple studies have demonstrated that the functionality (and relevance) of ACOD1 in infectious settings is specific to the bacterial species being studied. For example, myeloid cell-specific *Acod1* knockout mice are sensitive to *Mycobacterium tuberculosis* infection and have increased lung bacterial burden (92). *In-vitro* studies in *Acod1*-deficient bone marrow-derived macrophages also demonstrated enhanced replication of *Lactobacillus halophilus* (93). IFN-β or IFN-γ limits the intracellular growth of *Legionella pneumophila* by inducing the expression of ACOD1 and itaconate production *in vitro* and *in vivo* (93). Infection from *Salmonella* Typhimurium or *Mycobacterium avium* is also restricted by the induction of ACOD1 expression or itaconate production (31).

Although our study suggests a regulatory role for ACOD1 in environmental exposure-induced airway injury/inflammation, there are limitations. As this was an acute (one-time exposure) model of inflammatory agent-induced lung inflammation, we did not demonstrate consequences associated with chronic inflammatory lung disease, fibrosis development, or the associated fibrotic airway mechanics of changes in compliance and resistance. Future studies are warranted to investigate the role of ACOD1 in repeated environmental exposures. We also did not evaluate extrapulmonary organs (e.g., spleen, bone marrow, liver, kidney), apart from pulmonary draining lymph nodes, which may be necessary to clarify the systemic effect of ACOD1 in our animal model. There are also countless environmentally derived inflammatory agents in our exposome that could be responsible for adverse respiratory outcomes and should be explored including industrial chemicals (e.g., hydrogen sulfide, ammonia), heavy metals (e.g., cadmium, mercury), microbial agents (e.g., gram-positive peptidoglycan, fungal components), and other complex real-world exposures (e.g., burn pit exposures, air pollution, wildfire smoke).

In conclusion, ACOD1-deficient mice exhibited several decreased proinflammatory indicators, of those investigated, relative to ACOD1-sufficient mice. *Acod1* and proinflammatory transcripts (i.e., *Nos2*, *Ly6c1*) were also significantly upregulated in the murine monocyte/macrophage subpopulation representative of monocyte-derived macrophages (MDMs). Modulation of the ACOD1 immunometabolic axis could therefore prove beneficial via alteration of the MDM phenotype and function. Thus, future studies further characterizing the context-dependent regulatory effects of ACOD1 are necessary to optimally inform ACOD1/itaconate immunometabolic axis modulators to potentially elicit therapeutic benefit for at-risk persons.

## Data Availability

The datasets presented in this study can be found in online repositories. The names of the repository/repositories and accession number(s) can be found below: GSE267022 (GEO) and at https://doi.org/10.5281/zenodo.11060421 (Zenodo).

## Supplementary material

Supplementary Figure 1 Gating strategy for identification of non-debris, singlets, live CD45+ myeloid and lymphoid cells. For flow analysis, all panels were first gated as forward scatter-area (FSC-A) x side scatter-area (SSC-A) to omit debris, dead and/or apoptotic cells. This was followed by two single cell gates to omit doublets (FSC-A x FSC-height (H) and SSC-A x SSC-H), followed by a live/dead gate and then a CD45 gate to ensure removal of any additional dead or apoptotic cells and non-leukocytes. The CD45^+^ cells were gated on CD11c x Ly6G to select Ly6G^+^ neutrophils. Non-neutrophils were gated for B cells (CD19 x SSC gate). This was followed by non-B cells gated on CD11c x CD11b to select CD11c^+^CD11b^lo^ alveolar (Alv) macrophages (Mɸ), CD11c^+^CD11b^hi^ activated (Act) Mɸ, CD11c^int^CD11b^hi^ transitioning monocytes (Mono)-Mɸ, and CD11c^−^CD11b^hi^ monocytes (Mono). The negative or non-monocyte/macrophage populations were gated on CD3 x NK1.1 to select CD3^+^ T cells and CD3^−^NK1.1^+^ NK cells, and then a CD4 x CD8 gate to select CD3^+^CD4^+^ and CD3^+^CD8^+^ T cells.

Supplementary Figure 2 Gating strategy for characterization of pulmonary draining lymph node cellular composition. For flow analysis, all panels were first gated as forward scatter-area (FSC-A) x side scatter-area (SSC-A) to omit debris, dead and/or apoptotic cells. This was followed by two single cell gates to omit doublets (FSC-A x FSC-height (H) and SSC-A x SSC-H), followed by a live/dead gate and then a CD45 gate to ensure removal of any additional dead or apoptotic cells and non-leukocytes. The CD45+ cells were gated on CD11c x Ly6G to select Ly6G+ neutrophils. Non-neutrophils were gated for B cells (CD19 x SSC gate). This was followed by non-B cells gated on CD3 x CD24 to identify dendritic cells. Non-dendritic cells were gated for monocytes by selecting cells doubly positive for Ly6C x CD11b. Non-monocytes were then gated to distinguish macrophages (CD11c^+^CD11b^variable^). The negative gate that includes non-macrophages was gated by CD3 x NK1.1 to select CD3^+^ T cells and CD3^−^NK1.1^+^ NK cells, and then a CD4 x CD8 gate to select CD3^+^CD4^+^ and CD3^+^CD8^+^ T cells.

Supplementary Figure 3
*Acod1^-/-^* mice do not exhibit differences in LPS or ODE-induced lung inflammation or collagen 48 hours post-treatment. **(A)** Representative images from treatment groups stained by H&E. **(B)** Scatter plots with bars depict mean with SEM of semi-quantitative lung inflammatory score for each mouse. n=5 (CXN), n=19 (9 male and 10 female WT mice, LPS), n=18 (8 male and 10 female *Acod1^-/-^* mice, LPS), n=17 (7 male and 10 female WT mice, ODE), and n=19 (9 male and 10 female *Acod1^-/-^* mice, ODE). **(C)** Representative images from treatment groups stained by trichrome. **(D)** Scatter plot with bars depicts mean with SEM of integrate density of collagen quantified per each mouse. n=3 (CXN), n=6 (3 male and 3 female WT mice, LPS), n=5 (2 male and 3 female *Acod1^-/-^* mice, LPS), n=5 (2 male and 3 female WT mice, ODE), and n=6 (3 male and 3 female *Acod1^-/-^* mice, ODE). Statistical significance vs. CXN (##p<0.01).
