# Supplementary material for: Aconitate decarboxylase 1 mediates the acute airway inflammatory response to environmental exposures
Source: Front Immunol. 2024 Sep 16;15:1432334. doi: 10.3389/fimmu.2024.1432334 (PMC11439662; doi:10.3389/fimmu.2024.1432334)
Supplement: Supplementary file 1 [file Table1.docx]

| **Supplemental Table 1. Acute LPS-exposure induced lung and BALF responses in WT and *Acod1^-/-^* (vs. CXN) male and female mice.** | | | |
| --- | --- | --- | --- |
|  | **CXN** | **WT** | ***Acod1^-/-^*** |
| **BALF cells**, x10^5^ |  |  |  |
| Macrophages | 0.579 ± 0.0926 | 1.63 ± 0.255 | 1.57 ± 0.385 |
| Lymphocytes | 0.00696 ± 0.00338 | 0.318 ± 0.0842 | 0.233 ± 0.0588 |
| **Lung tissue cells**, x10^5^ |  |  |  |
| Total Cells | 3.83 ± 0.228 | 29.7 ± 2.84^####^ | 23.6 ± 2.27^##^ |
| CD8^+^ T Cells | 0.163 ± 0.0141 | 0.977 ± 0.143^##^ | 1.11 ± 0.162^##^ |
| NK Cells | 0.226 ± 0.00814 | 0.503 ± 0.0734 | 0.580 ± 0.0943 |
| Alveolar Macrophages | 0.934 ± 0.0790 | 0.0737 ± 0.0164^###^ | 0.0701 ± 0.0115^##^ |
| Activated Macrophages | 0.0133 ± 0.00138 | 0.974 ± 0.138^##^ | 0.868 ± 0.106^##^ |
| Monocyte-Macrophages | 0.160 ± 0.0121 | 2.88 ± 0.418^###^ | 2.39 ± 0.411^##^ |
| **BALF mediators**, pg/ml |  |  |  |
| IL-10 | 70.2 ± 21.2 | 38.7 ± 7.35 | 58.8 ± 9.17 |
| **Lung mediators** |  |  |  |
| IL-10 (pg/ml) | 626 ± 115 | 1603 ± 226 | 2077 ± 321^#^ |
| MMP-3 (ng/ml) | 1.08 ± 0.273 | 33.3 ± 7.98 | 32.5 ± 9.34 |
| TGF-β (pg/ml) | 82.14 ± 19.2 | 233 ± 22.8^#^ | 262 ± 36.2^##^ |
| IFN-γ (pg/ml) | 24.7 ± 1.91 | 229 ± 60.5^##^ | 182 ± 35.3^##^ |
| **Baseline lung function** |  |  |  |
| Dynamic compliance (mL/cm H_2_O) | 0.017 ± 0.0008 | 0.015 ± 0.0006 | 0.013 ± 0.001 |
| Statistical difference vs. CXN (^#^p<0.05, ^##^p<0.01, ^###^p<0.001, ^####^p<0.001)  n=2-5 (CXN), n=19 (9 male and 10 female WT mice), and n=16-18 (6-8 male and 10 female *Acod1^-/-^* mice)  n=6 male mice per group for baseline lung function assessment | | | |
