# Supplementary material for: Aconitate decarboxylase 1 mediates the acute airway inflammatory response to environmental exposures
Source: Front Immunol. 2024 Sep 16;15:1432334. doi: 10.3389/fimmu.2024.1432334 (PMC11439662; doi:10.3389/fimmu.2024.1432334)
Supplement: Supplementary file 2 [file Table2.docx]

| **Supplemental Table 2. Acute ODE-exposure induced lung and BALF responses in WT and *Acod1^-/-^* (vs. CXN) male and female mice.** | | | |
| --- | --- | --- | --- |
|  | **CXN** | **WT** | ***Acod1^-/-^*** |
| **BALF cells**, x10^5^ |  |  |  |
| Macrophages | 0.579 ± 0.0926 | 1.57 ± 0.218^#^ | 1.62 ± 0.305 |
| Lymphocytes | 0.00696 ± 0.00338 | 0.106 ± 0.0400 | 0.189 ± 0.0458^#^ |
| **Lung tissue cells**, x10^5^ |  |  |  |
| Total Cells | 3.83 ± 0.228 | 12.6 ± 1.22^##^ | 9.06 ± 0.734^#^ |
| CD8^+^ T Cells | 0.163 ± 0.0141 | 0.650 ± 0.0615^###^ | 0.558 ± 0.0525^##^ |
| NK Cells | 0.226 ± 0.00814 | 0.439 ± 0.0370^#^ | 0.432 ± 0.0347^#^ |
| Alveolar Macrophages | 0.934 ± 0.0790 | 0.421 ± 0.0561^####^ | 0.340 ± 0.173^####^ |
| Activated Macrophages | 0.0133 ± 0.00138 | 1.89 ± 0.271^###^ | 1.52 ± 0.176^##^ |
| Monocyte-Macrophages | 0.160 ± 0.0121 | 1.58 ± 0.215^###^ | 0.997 ± 0.131^#^ |
| **BALF mediators**, pg/ml |  |  |  |
| IL-10 | 70.2 ± 21.2 | 72.3 ± 15.5 | 111 ± 19.4 |
| **Lung mediators**, pg/ml |  |  |  |
| IL-10 | 626 ± 115 | 1678 ± 202 | 1747 ± 242 |
| MMP-3 (ng/ml) | 1.08 ± 0.273 | 53.1 ± 4.10^#^ | 44.7 ± 4.70 |
| TGF-β (pg/ml) | 82.1 ± 19.2 | 357 ± 50.4^#^ | 336 ± 36.4^#^ |
| IFN-γ (pg/ml) | 24.7 ± 1.91 | 26.5 ± 3.30 | 32.0 ± 3.34 |
| Statistical difference vs. CXN (^#^p<0.05, ^##^p<0.01, ^###^p<0.001, ^####^p<0.001)  n=2-5 (CXN), n=14-17 (7 male and 7-10 female WT mice), and n=19 (9 male and 10 female *Acod1^-/-^* mice) | | | |
