# Supplementary material for: Aconitate decarboxylase 1 mediates the acute airway inflammatory response to environmental exposures
Source: Front Immunol. 2024 Sep 16;15:1432334. doi: 10.3389/fimmu.2024.1432334 (PMC11439662; doi:10.3389/fimmu.2024.1432334)
Supplement: Supplementary file 3 [file Table3.docx]

| **Supplemental Table 3. WT and *Acod1^-/-^* mice do not demonstrate significant differences in pulmonary draining lymph node cellular composition following a one-time, lung-delivered LPS exposure.** | | |
| --- | --- | --- |
|  | **WT** | ***Acod1^-/-^*** |
| **Lymph node**, % of CD45^+^ cells |  |  |
| Neutrophils | 2.44 ± 0.70 | 0.93 ± 0.32 |
| B Cells | 28.7 ± 8.42 | 19.3 ± 6.70 |
| CD4^+^ T Cells | 14.7 ± 3.24 | 18.6 ± 3.33 |
| CD8^+^ T Cells | 9.54 ± 4.31 | 14.7 ± 4.58 |
| NK Cells | 5.14 ± 0.67 | 3.44 ± 1.26 |
| Monocytes | 4.44 ± 1.44 | 1.48 ± 0.65 |
| Macrophages | 0.14 ± 0.01 | 0.10 ± 0.03 |
| Dendritic Cells | 21.0 ± 9.92 | 27.6 ± 10.9 |
| n=5 (3 male and 2 female WT mice) and n=5 (3 male and 2 female *Acod1^-/-^* mice) | | |
