# Supplementary figures and images for: Aconitate decarboxylase 1 mediates the acute airway inflammatory response to environmental exposures
Source: Front Immunol. 2024 Sep 16;15:1432334. doi: 10.3389/fimmu.2024.1432334 (PMC11439662; doi:10.3389/fimmu.2024.1432334)

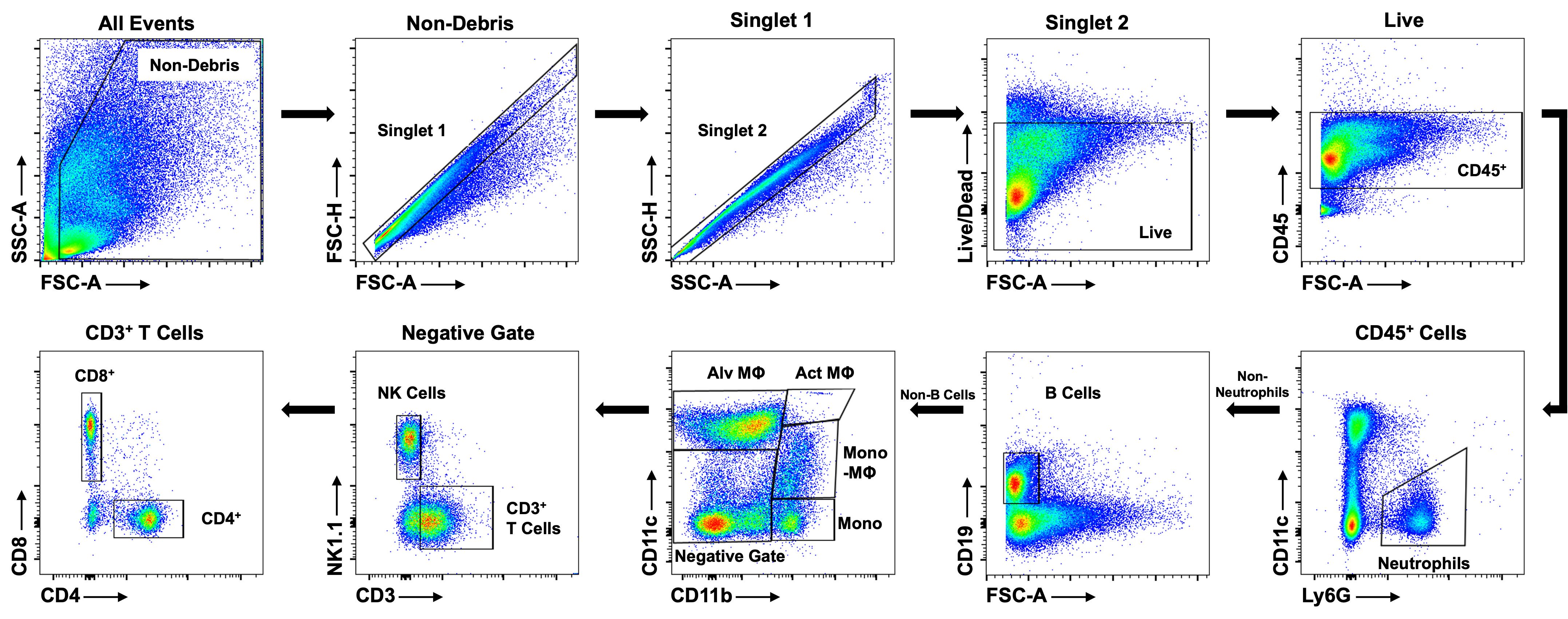

Supplement: Supplementary Figure 1 — Gating strategy for identification of non-debris, singlets, live CD45+ myeloid and lymphoid cells. For flow analysis, all panels were first gated as forward scatter-area (FSC-A) x side scatter-area (SSC-A) to omit debris, dead and/or apoptotic cells. This was followed by two single cell gates to omit doublets (FSC-A x FSC-height (H) and SSC-A x SSC-H), followed by a live/dead gate and then a CD45 gate to ensure removal of any additional dead or apoptotic cells and non-leukocytes. The CD45+ cells were gated on CD11c x Ly6G to select Ly6G+ neutrophils. Non-neutrophils were gated for B cells (CD19 x SSC gate). This was followed by non-B cells gated on CD11c x CD11b to select CD11c+CD11blo alveolar (Alv) macrophages (Mɸ), CD11c+CD11bhi activated (Act) Mɸ, CD11cintCD11bhi transitioning monocytes (Mono)-Mɸ, and CD11c−CD11bhi monocytes (Mono). The negative or non-monocyte/macrophage populations were gated on CD3 x NK1.1 to select CD3+ T cells and CD3−NK1.1+ NK cells, and then a CD4 x CD8 gate to select CD3+CD4+ and CD3+CD8+ T cells. [file Image1.jpeg]

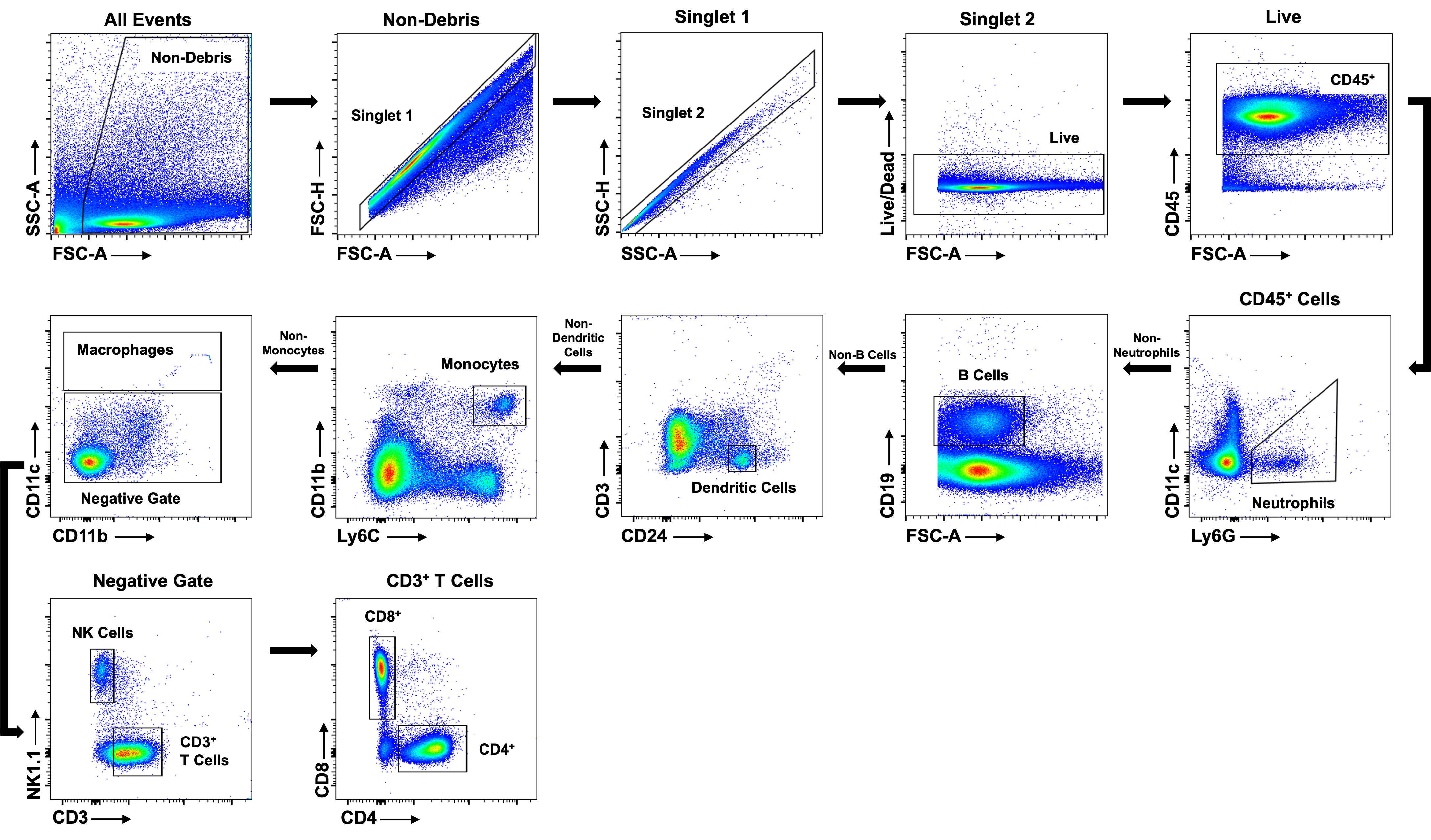

Supplement: Supplementary Figure 2 — Gating strategy for characterization of pulmonary draining lymph node cellular composition. For flow analysis, all panels were first gated as forward scatter-area (FSC-A) x side scatter-area (SSC-A) to omit debris, dead and/or apoptotic cells. This was followed by two single cell gates to omit doublets (FSC-A x FSC-height (H) and SSC-A x SSC-H), followed by a live/dead gate and then a CD45 gate to ensure removal of any additional dead or apoptotic cells and non-leukocytes. The CD45+ cells were gated on CD11c x Ly6G to select Ly6G+ neutrophils. Non-neutrophils were gated for B cells (CD19 x SSC gate). This was followed by non-B cells gated on CD3 x CD24 to identify dendritic cells. Non-dendritic cells were gated for monocytes by selecting cells doubly positive for Ly6C x CD11b. Non-monocytes were then gated to distinguish macrophages (CD11c+CD11bvariable). The negative gate that includes non-macrophages was gated by CD3 x NK1.1 to select CD3+ T cells and CD3−NK1.1+ NK cells, and then a CD4 x CD8 gate to select CD3+CD4+ and CD3+CD8+ T cells. [file Image2.jpeg]

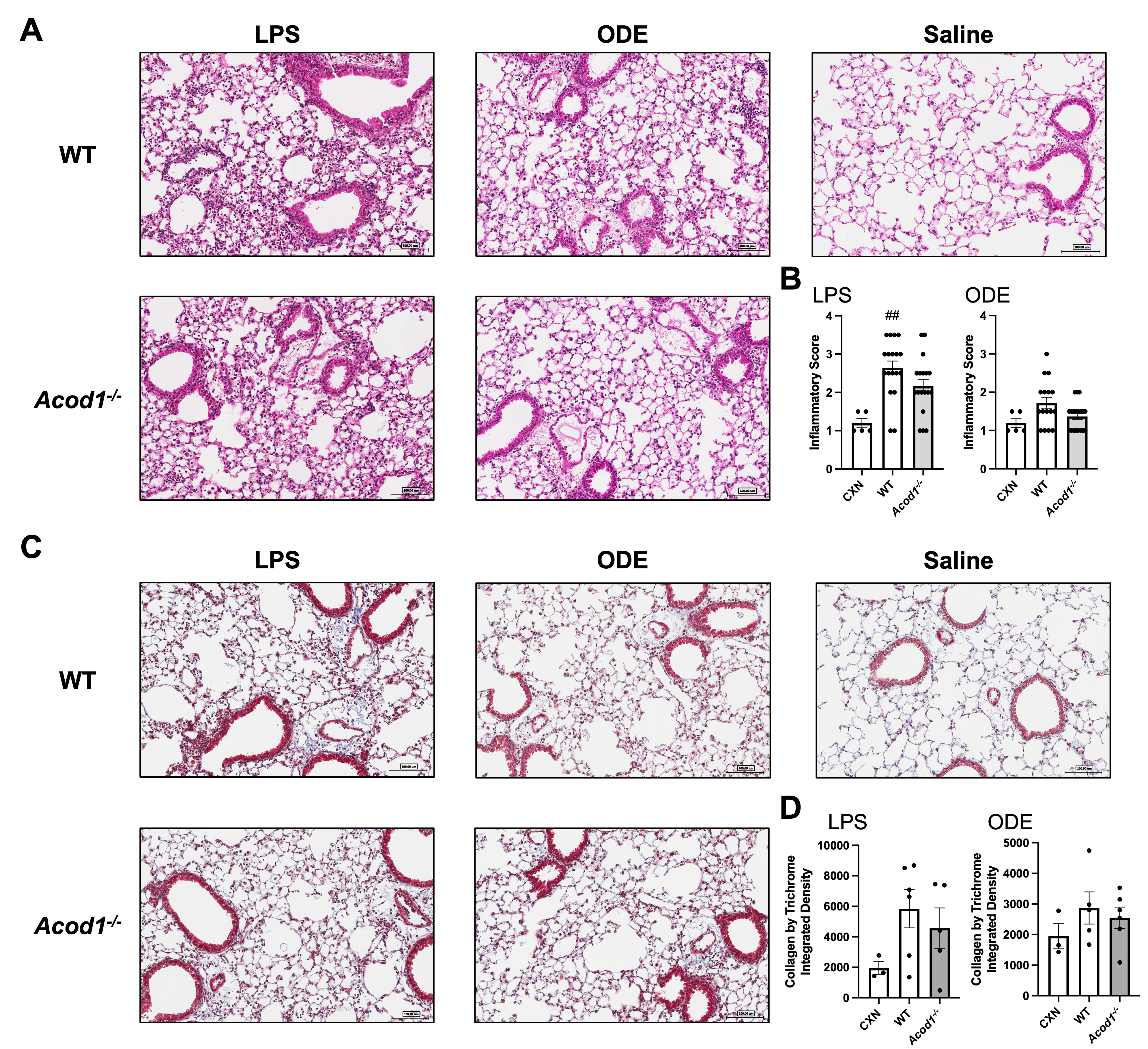

Supplement: Supplementary Figure 3 — Acod1-/- mice do not exhibit differences in LPS or ODE-induced lung inflammation or collagen 48 hours post-treatment. (A) Representative images from treatment groups stained by H&E. (B) Scatter plots with bars depict mean with SEM of semi-quantitative lung inflammatory score for each mouse. n=5 (CXN), n=19 (9 male and 10 female WT mice, LPS), n=18 (8 male and 10 female Acod1-/- mice, LPS), n=17 (7 male and 10 female WT mice, ODE), and n=19 (9 male and 10 female Acod1-/- mice, ODE). (C) Representative images from treatment groups stained by trichrome. (D) Scatter plot with bars depicts mean with SEM of integrate density of collagen quantified per each mouse. n=3 (CXN), n=6 (3 male and 3 female WT mice, LPS), n=5 (2 male and 3 female Acod1-/- mice, LPS), n=5 (2 male and 3 female WT mice, ODE), and n=6 (3 male and 3 female Acod1-/- mice, ODE). Statistical significance vs. CXN (##p<0.01). [file Image3.jpeg]
